# Temporal-spatial distribution characteristics and associated socioeconomic factors of visiting frequency for rural patients with hypertension in Fujian Province, Southeast China

**DOI:** 10.1186/s12889-024-18113-9

**Published:** 2024-03-01

**Authors:** Rong Fu, Zhi Huang, Yulan Lin, Xuwei Tang, Zhenquan Zheng, Zhijian Hu

**Affiliations:** 1 Department of Epidemiology and Health Statistics, Fujian Provincial Key Laboratory of Environmental Factors and Cancer, School of Public Health, Fujian Medical University, 1 Xuefu north Road, Fuzhou, Fujian 350122 P.R. China; 2 School of Health Management, Fujian Medical University, Fuzhou, 350122 China

**Keywords:** Visiting frequency, Follow up, Temporal-spatial distribution, Hypertension, China

## Abstract

**Background:**

Regular follow-up and medication can effectively reduce the risk of adverse outcomes for patients with hypertension. This study aimed to explore the temporal-spatial distribution characteristics and associated socioeconomic factors of visiting frequency for rural patients with hypertension in Fujian province from 2011 to 2016.

**Methods:**

The medical records of patients with hypertension were abstracted from the database of New Rural Cooperative Medical Scheme. Geographically and temporally weighted regression model was used to analyze the associations between percentage of patients whose visiting frequency ≥ 4 times within a year and seven socioeconomic factors at the county level.

**Results:**

The visiting rate of rural patients with hypertension was 0.79%, 1.27%, 1.87%, 2.29%, 2.78%, 3.43% over the six-year study period, respectively. The percentage of patients whose visiting frequency ≥ 4 times within a year gradually increased over time and the percentage ranged from 61 to 80% in a half of the counties by 2016. In general, there was positive association between Gross Domestic Product per capita and the percentage of patients whose visiting frequency ≥ 4 times within a year. The percentage of female patients, percentage of patients who aged ≥ 60 years, percentage of low-income patients, carbon emission intensity, percentage of savings and number of health technicians per 10,000 persons were negatively correlated with the percentage of patients whose visiting frequency ≥ 4 times within a year in most of counties of Fujian Province. In the sensitivity analysis, the percentage of outpatients whose visiting frequency ≥ 4 times within a year was higher than that of all patients. There was positive association between percentage of outpatients who aged ≥ 60 years and the percentage of outpatients whose visiting frequency ≥ 4 times.

**Conclusions:**

The visiting rate and the visiting frequency within a year for rural patients with hypertension in Fujian province need to be improved. Female and elderly patients should be the focus of health management. Effectively implementing the family doctor services, providing several kinds of free antihypertensive drugs, improving energy utilization efficiency and reasonably allocating the health resources may be the effective strategies to improve the follow-up compliance of patients.

**Supplementary Information:**

The online version contains supplementary material available at 10.1186/s12889-024-18113-9.

## Introduction

Hypertension is one of the major public health problems in the world and associated with a substantial disease burden [1–3]. The prevalence of hypertension (blood pressure level ≥ 140/90 mm Hg) was 23.2% among Chinese adult population ≥ 18 years of age in 2012–2015 and 32.0% among American adults aged 20 years and older in 2013–2016 [4, 5]. The direct economic burden caused by hypertension amounted to 210.3 billion yuan, accounting for 6.61% of China’s total health expenditures in 2013 [6]. It was predicted that the direct and indirect costs of hypertension in the U.S. population would increase to 200 billion and 40 billion dollars by 2030, respectively [2, 7]. The leading risk factor globally for attributable deaths was high systolic blood pressure (BP) which accounted for 10.8 million deaths (19.2% of all deaths) in 2019 [8].

Although there is no cure for hypertension, regular follow-up and medication can keep most patients’ BP at a safe level [1, 9]. Effective control of hypertension is an important way to reduce the morbidity and mortality of chronic diseases such as cardiovascular diseases, stroke, kidney diseases and so on [10]. The State Council of China deepened the healthcare system reform and launched the National Basic Public Health Service Project in 2009, under which urban and rural primary medical institutions provided health management services for patients with hypertension. Many policies and strategies, such as risk factor intervention, health education and health management, were made to prevent and control the prevalence of hypertension [11, 12]. However, the rate of patients who were aware of their condition, received antihypertensive treatment, and controlled their condition in China was < 50%, < 40% and < 10%, respectively [13–15].

Outpatient service was the most common use of health services for patients with hypertension. It was reported that there were substitution effects of outpatient services on inpatient services [16]. The increase of outpatient visits would significantly reduce excessive demands for inpatient service and inpatient expenditures [16, 17]. The guidelines for prevention and treatment of hypertension suggested that the patients who achieved targeted BP level should be followed up once in 3 months and once in 2–4 weeks for those who did not achieve [18]. In other words, the patients with hypertension should see a doctor at least four times within a year. Regular follow-up was the key link to implement effective strategies to prevent and control hypertension, and was considered to greatly increase the awareness, treatment and control of hypertension.

Substantial studies had reported the awareness, treatment and control of hypertension [13–15], but there was little knowledge about the visiting frequency for patients with hypertension. Health service utilization for hypertension often depended on the accessibility of health service and socioeconomic resources, which always had geographically heterogeneous. Therefore, this study described the temporal-spatial distribution characteristics of visiting frequency for patients with hypertension and incorporated a temporal-spatial perspective to analyze the associated socioeconomic factors of visiting frequency in Fujian province from 2011 to 2016. It aimed to supplement the effective strategies to decrease disease burden and increase the awareness and control of hypertension on the existing basis.

## Methods

### Data source

New Rural Cooperative Medical Scheme (NRCMS) was promulgated to improve the health security and reduce the disease burden for rural residents in 2003 [19]. The polices related to NRCMS were expanded to the whole of Fujian province in 2007. The participation rate of NRCMS increased from 92.11% in 2011 to 97.94% in 2016 in Fujian province (Supplementary Table 1). Individual-level visiting data in this study was extracted from the database of NRCMS in Fujian province from 2011 to 2016. The database managers removed the names, addresses and telephone numbers before abstracting the data to protect patient privacy. Every patient was identified by a unique NRCMS card number.

### Study population

Hypertension, which was diagnosed by doctors, was defined as an average systolic blood pressure (BP) ≥ 140 mmHg and/or diastolic BP ≥ 90 mmHg measured three times on different days. The medical records of patients whose diagnoses included “hypertension” were screened from the outpatient and inpatient database of NRCMS and then the medical records in the two databases were merged. The diagnoses of inpatients were relatively complex and generally included etiology or complications of hypertension in addition to hypertension. Patient variables included NRCMS card number, gender, age, residential county, low-income or not, total medical expenditures, reimbursement expenses and visiting type (outpatient, inpatient or both). The cumulative total medical expenditures, cumulative reimbursement expenses and visiting frequency of each patient within a natural year were calculated using NRCMS card number as key variable. Thus, there was only one record per patient in each natural year in the final analysis dataset. The number of rural patients with hypertension who visited in all medical institutions of Fujian province ranged from 113,192 in 2011 to 473,341 in 2016.

### Socioeconomic factors

Seven socioeconomic factors at the county-level were used to analyze their associations with visiting frequency for patients with hypertension, including: (1) Percentage of female patients (%): the number of female patients divided by the total number of patients; (2) Percentage of patients who aged ≥ 60 years (%): the number of patients who aged ≥ 60 years divided by the total number of patients; (3) Percentage of low-income patients (%): the number of low-income patients divided by the total number of patients; (4) Gross Domestic Product (GDP) per capita (10,000 yuan per capita): the ratio of GDP to the average population; (5) Carbon emission intensity (ton per 10,000 yuan): the amount of carbon dioxide emitted to generate a unit of GDP; (6) Percentage of savings (%): the difference between per capita disposable income and per capita consumption expenditure divided by per capita disposable income; (7) Number of health technicians (HT) per 10,000 persons: the ratio of the number of HT to the average population. Factor (1), (2) and (3) were calculated using the database of NRCMS for patients with hypertension. The data of the other factors was extracted from Fujian Provincial Bureau of Statistics (Accessible at https://tjj.fujian.gov.cn/xxgk/ndsj/). Carbon emissions at the county-level was estimated by Chen et al. using a particle swarm optimization-back propagation (PSO-BP) algorithm [20].

### Statistical analysis

Number and percentage were calculated for categorical variables. Mean, standard deviation, minimum, quartiles and maximum were calculated for numerical variables. Lorenz curve (cumulative proportion of patients as horizontal axis and cumulative proportion of expenditures as vertical axis) and Gini coefficient were used to assess the clustering of medical expenditures for patients with hypertension. If Gini coefficient was greater than 0.5, the clustering was remarkable. The percentage of visiting frequency (1, 2, 3 and ≥ 4 times) within a year of patients with hypertension was calculated for each county. The global Moran’s I and Anselin local Moran’s I were used to assess the spatial autocorrelation of percentage of visiting frequency ≥ 4 times within a year in Fujian Province. General linear model (GLM) was used to preliminarily assess the associations between the percentage of patients whose visiting frequency ≥ 4 times within a year and seven socioeconomic factors, and test the multicollinearity among socioeconomic factors. If variance inflation factor (VIF) was greater than 10, there was multicollinearity. Geographically and temporally weighted regression model (GTWRM) can deal with spatial and temporal heterogeneity simultaneously [21]. This study reassessed the associations between the percentage of patients whose visiting frequency ≥ 4 times within a year and seven socioeconomic factors using GTWRM. Considering the follow-up compliance was different between outpatients and inpatients, sensitivity analysis was conducted excluding inpatients with hypertension. The statistics of dependent and explanatory variables in GTWRM were presented in Supplementary Table 2, including mean, standard deviation, minimum, percentile 25, median, percentile 75 and maximum. Data cleansing, descriptive statistics and GLM were conducted using SPSS Statistics 26 (IBM China Company Limited). Temporal-spatial distribution characteristics of the percentage of patients whose visiting frequency ≥ 4 times within a year, spatial autocorrelation analysis and GTWRM were conducted using ArcGIS 10.5 (ESRI China Information Technology Company Limited). The public version of China’s 2021 basic geographic information data was obtained from the National Catalogue Service for Geographic Information website in China (Accessible at https://www.webmap.cn/commres.do?method=result100W). This map is authorized by the Chinese Ministry of Natural Resources and made freely available to the public which does not involve any copyright issues. The acquired map data, after conversion to Shapefile (shp) format, was used for drawing the maps with ArcGIS 10.5 in this study.

## Results

### Demographic characteristics

The number of rural patients with hypertension who visited in medical institutions increased from 113,192 in 2011 to 473,341 in 2016. The visiting rate was 0.79%, 1.27%, 1.87%, 2.29%, 2.78%, 3.43% over the six-year study period, respectively (Supplementary Table 1). Of all the patients, about 60% were female and 65% were aged ≥ 60 years. Around 40% of patients lived in coastal areas. The proportion of low-income patients was approximately 2%. The proportion of patients who visited only in an outpatient setting within a natural year increased from 51.2% in 2011 to 86.4% in 2016. Correspondingly, the proportion of hospitalized patients decreased year by year from 2011 to 2016 (Table 1).

**Table 1:** Demographic characteristics and expenses of study population from 2011 to 2016

Characteristic	2011	2012	2013	2014	2015	2016
Gender, n (%)						
Male	45,508 (40.2)	73,960 (40.5)	105,852 (41.1)	133,231 (41.4)	160,488 (41.3)	195,724 (41.3)
Female	67,684 (59.8)	108,581 (59.5)	151,726 (58.9)	188,661 (58.6)	228,401 (58.7)	277,617 (58.7)
Age, n (%)						
< 60 years	39,741 (35.1)	65,352 (35.8)	91,872 (35.7)	112,998 (35.1)	128,170 (33.0)	150,486 (31.8)
≥ 60 years	73,451 (64.9)	117,189 (64.2)	165,706 (64.3)	208,894 (64.9)	260,719 (67.0)	322,855 (68.2)
Domicile place, n (%)						
Inland	70,497 (62.3)	120,510 (66.0)	161,815 (62.8)	192,914 (59.9)	215,376 (55.4)	258,290 (54.6)
Coastland	42,695 (37.7)	62,031 (34.0)	95,763 (37.2)	128,978 (40.1)	173,513 (44.6)	215,051 (45.4)
Low-income, n (%)						
No	110,614 (97.7)	179,010 (98.1)	252,865 (98.2)	316,141 (98.2)	382,463 (98.3)	465,474 (98.3)
Yes	2,578 (2.3)	3,531 (1.9)	4,713 (1.8)	5,751 (1.8)	6,426 (1.7)	7,867 (1.7)
Visiting type, n (%)						
Outpatient	58,002 (51.2)	110,289 (60.4)	178,127 (69.2)	253,007 (78.6)	326,432 (83.9)	408,774 (86.4)
Inpatient	49,946 (44.1)	61,393 (33.6)	61,717 (24)	49,738 (15.5)	42,117 (10.8)	40,638 (8.6)
Both	5,244 (4.6)	10,859 (5.9)	17,734 (6.9)	19,147 (5.9)	20,340 (5.2)	23,929 (5.1)
Per capita total medical expenditures, yuan	2,176.6	1,889.8	1,837.0	1,679.1	1,538.7	1,498.0
Per capita reimbursement expenses, yuan	1,151.3	1,163.2	1,129.1	947.9	888.0	926.9
Reimbursement ratio ^a^, %	52.9	61.6	61.5	56.5	57.7	61.9
Per capita disposable income ^b^, yuan	8,779	9,967	11,405	12,650	13,793	14,999
Per capita out-of-pocket expenses ^c^, yuan	1,025.3	726.6	707.9	731.2	650.7	571.1
Out-of-pocket ratio ^d^, %	11.7	7.3	6.2	5.7	4.7	3.8
Gini coefficient	0.556	0.587	0.606	0.614	0.601	0.592

^a^: “Reimbursement ratio” was equal to “per capita reimbursement expenses” divided by “per capita total medical expenditures”; ^b^: Extracted from Fujian Provincial Bureau of Statistics (Accessible at https://tjj.fujian.gov.cn/xxgk/ndsj/); ^c^: “Per capita out-of-pocket expenses” was equal to “per capita total medical expenditures” minus “per capita reimbursement expenses”; ^d^: “Out-of-pocket ratio” was equal to “per capita out-of-pocket expenses” divided by “per capita disposable income”

### Medical expenses

The per capita total medical expenditures of rural patients with hypertension decreased from 2,176.6 yuan in 2011 to 1,498.0 yuan in 2016. Although the per capita reimbursement expenses also reduced, reimbursement ratio increased by 9% over the six-year study period. Out-of-pocket ratio, which was equal to per capita out-of-pocket expenses divided by per capita disposable income, decreased from 11.7% in 2011 to 3.8% in 2016 (Table 1). However, the Gini coefficients were all greater than 0.5 in all years and showed an increasing trend (Table 1 and Supplementary Fig. 1).

### Visiting frequency within a year

**Fig. 1:**
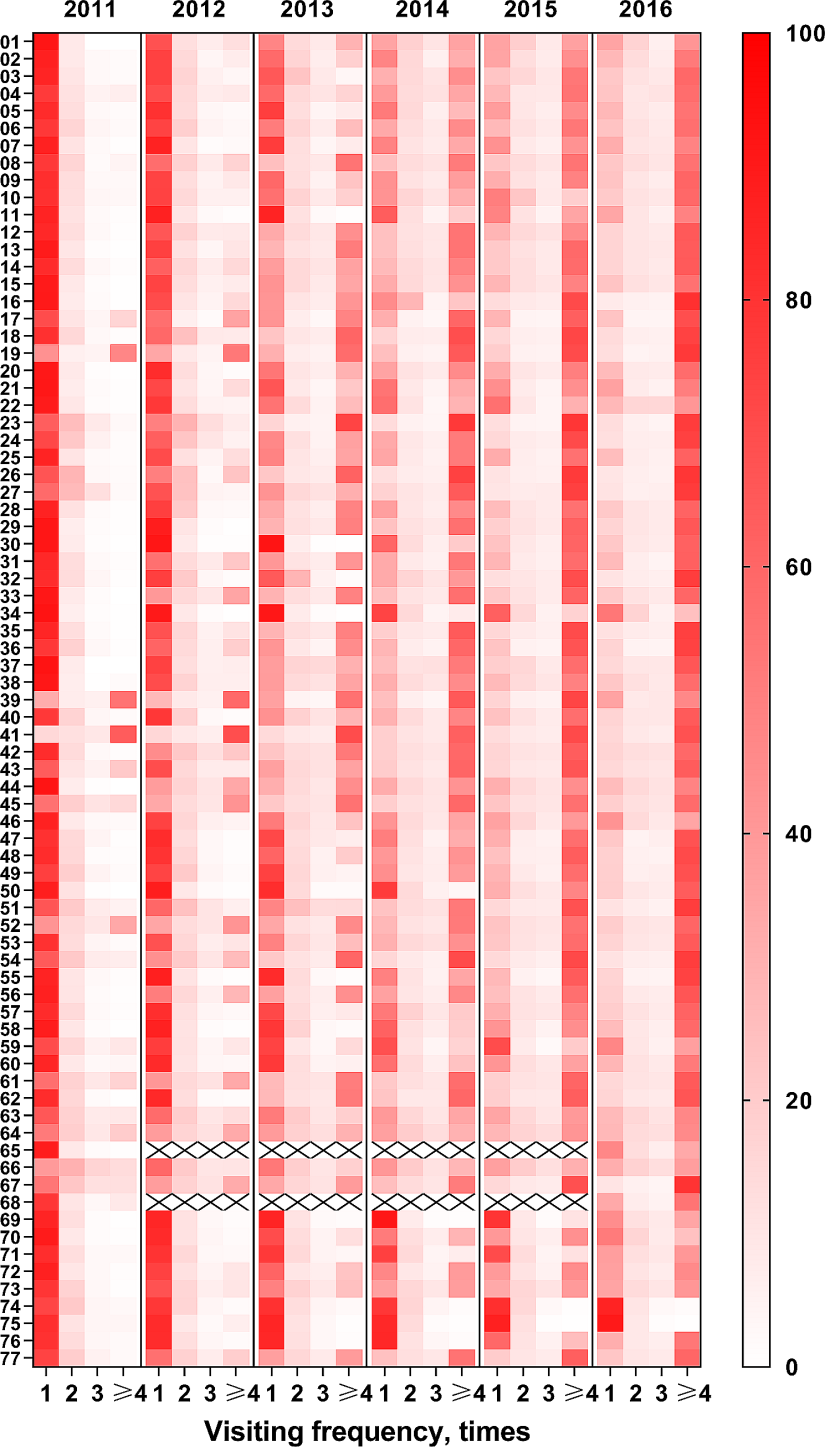
Percentage of visiting frequency within a year for rural patients with hypertension in 77 counties from 2011 to 2016. Each line represents a county. Each column represents visiting frequency (times). The redder the color, the higher the proportion. ×: no data

Figure 1 displayed the percentage of visiting frequency for rural patients with hypertension in 77 counties from 2011 to 2016. Each line represents a county and each column represents visiting frequency (times). The redder the color, the higher the percentage. In almost all counties, most of rural patients with hypertension saw a doctor only once in 2011 and 2012. Since 2013, the percentage of patients whose visiting frequency equal to one time gradually decreased while the percentage of patients whose visiting frequency ≥ 4 times gradually increased. By 2016, more than 50% of patients with hypertension saw a doctor at least 4 times in most of counties.

### Temporal-spatial distribution characteristics of percentage of patients whose visiting frequency ≥ 4 times within a year

**Fig. 2:**
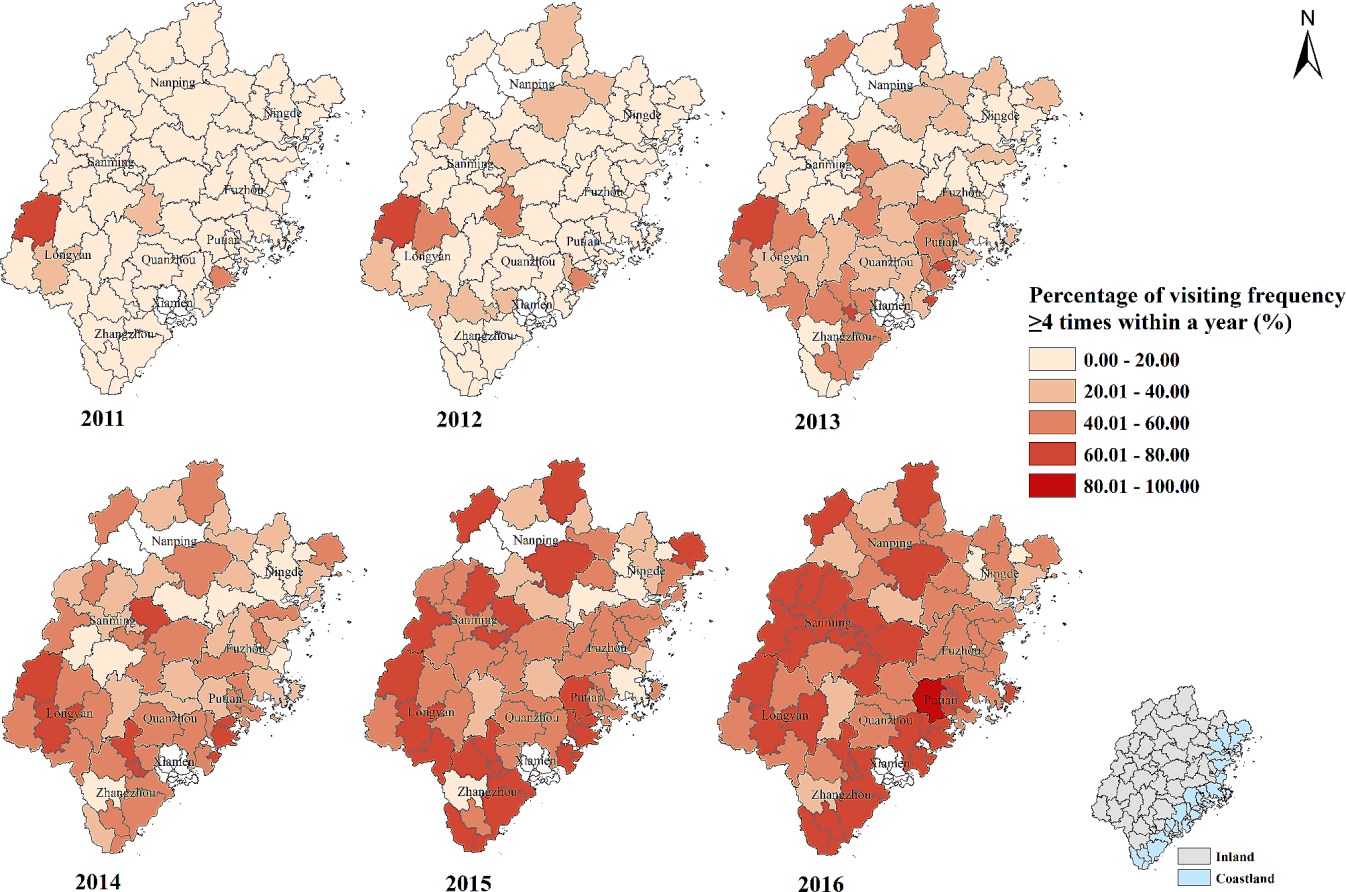
Percentage of visiting frequency ≥ 4 times within a year for rural patients with hypertension in Fujian province from 2011 to 2016. The redder the color, the higher the percentage. White color: no data

The percentage of patients whose visiting frequency ≥ 4 times were less than 20% in most of counties in 2011 and 2012. The percentage began to increase in southern and western areas and ranged from 41 to 60% in a third of the counties in 2013. The increase of the percentage in northern and eastern areas was also found since 2014. By 2016, the counties with the percentage between 61% and 80% were mainly concentrated in Putian, Quanzhou, Zhangzhou, Longyan, Sanming and Nanping city (Fig. 2). Except for 2011 and 2012, the percentage all showed spatial autocorrelation in other years (Supplementary Table 3). Overall, the percentage was presented low-low cluster in Ningde city and high-high cluster in Quanzhou, Zhangzhou and Longyan city (Supplementary Fig. 2).

### Associated socioeconomic factors of percentage of patients whose visiting frequency ≥ 4 times within a year

**Fig. 3:**
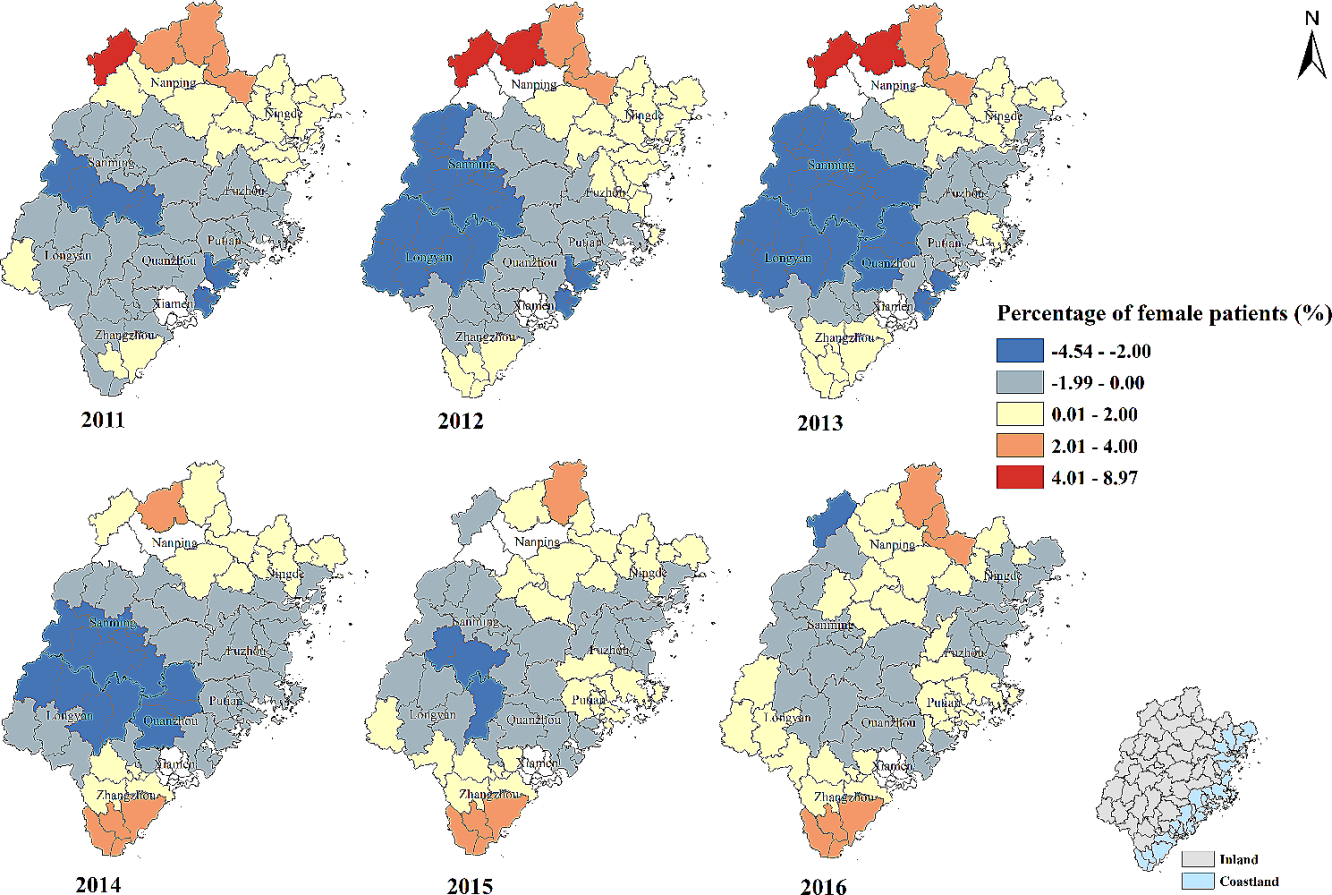
Temporal-spatial distribution of regression coefficients for “Percentage of female patients (%)” in geographically and temporally weighted regression model. White color: no data

**Fig. 4:**
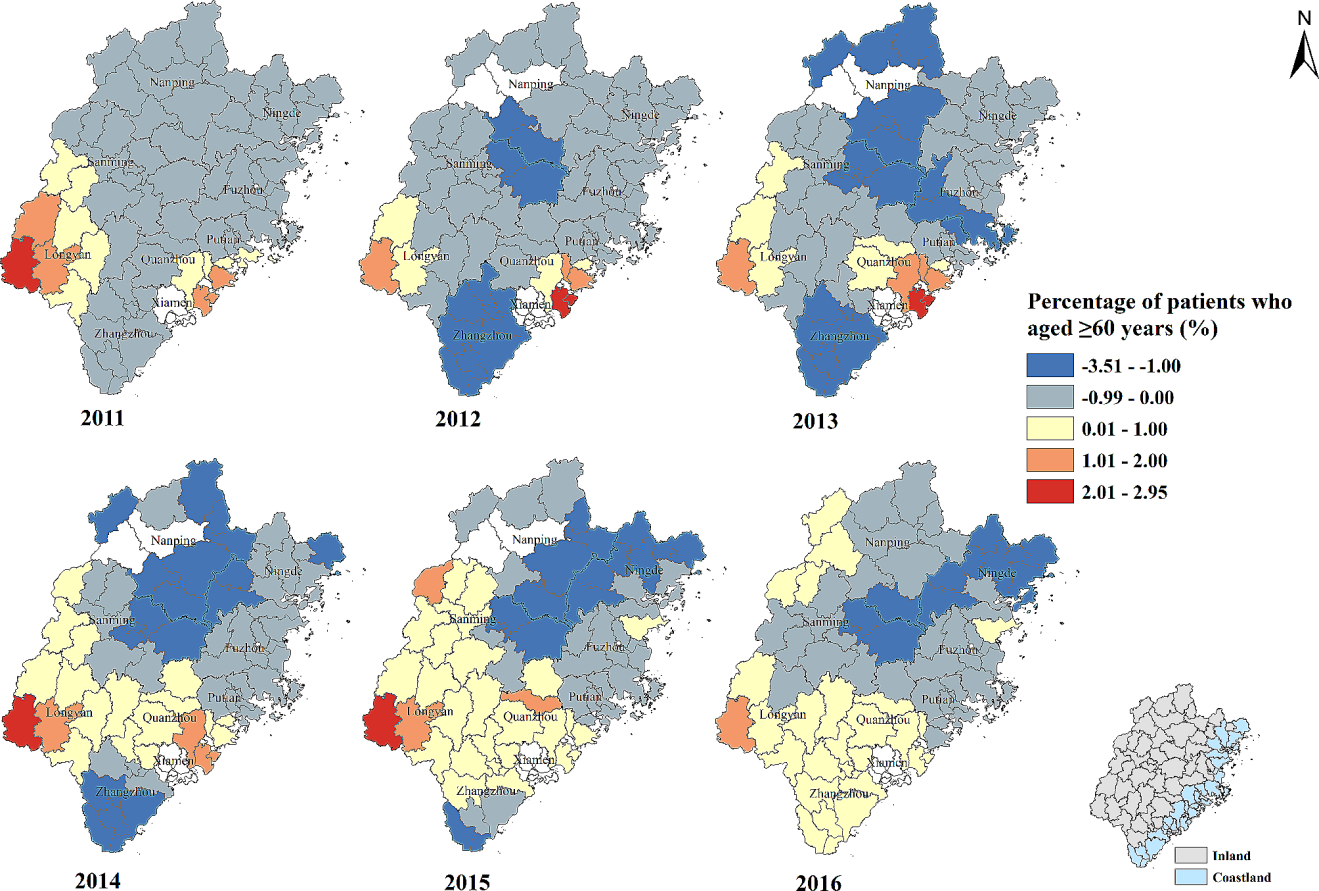
Temporal-spatial distribution of regression coefficients for “Percentage of patients who aged ≥ 60 years (%)” in geographically and temporally weighted regression model. White color: no data

**Fig. 5:**
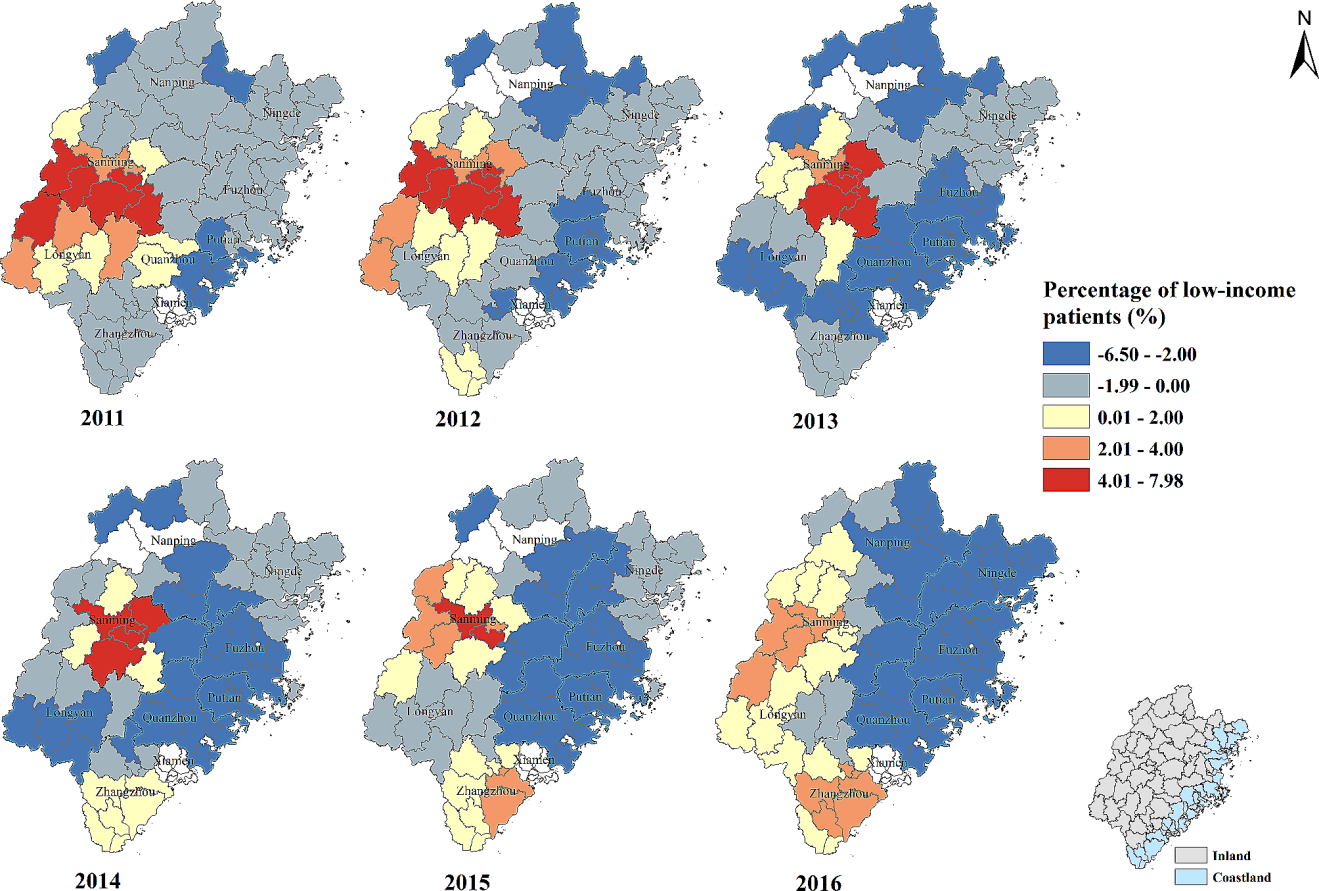
Temporal-spatial distribution of regression coefficients for “Percentage of low-income patients (%)” in geographically and temporally weighted regression model. White color: no data

**Fig. 6:**
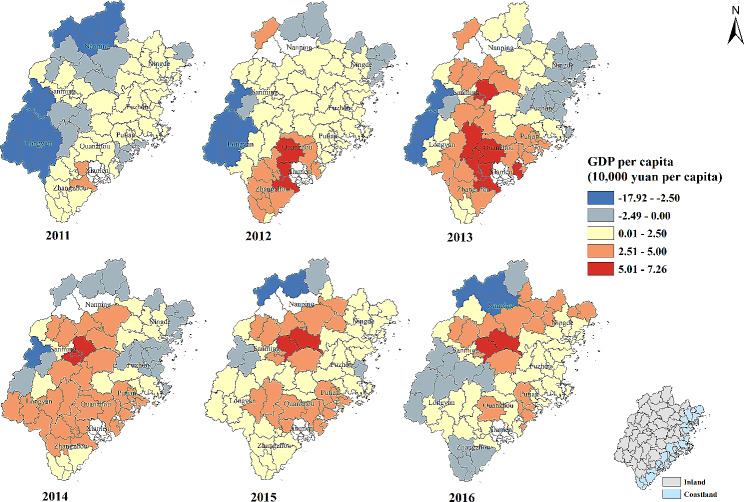
Temporal-spatial distribution of regression coefficients for “GDP per capita (10,000 yuan per capita)” in geographically and temporally weighted regression model. GDP: Gross Domestic Product. White color: no data

**Fig. 7:**
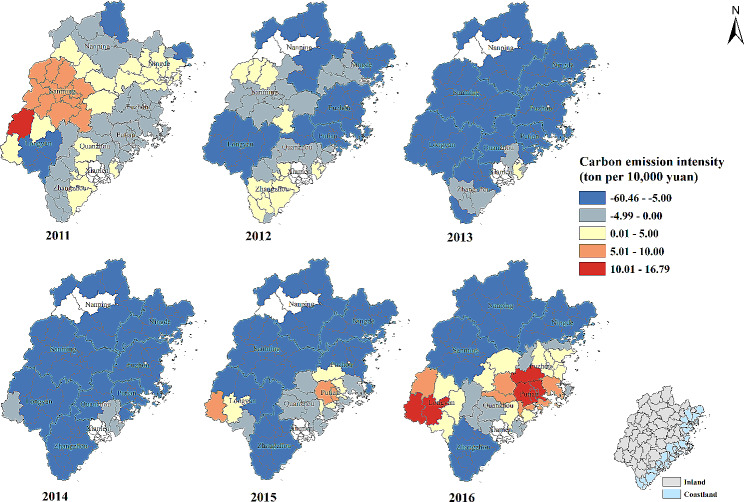
Temporal-spatial distribution of regression coefficients for “Carbon emission intensity (ton per 10,000 yuan)” in geographically and temporally weighted regression model. White color: no data

**Fig. 8:**
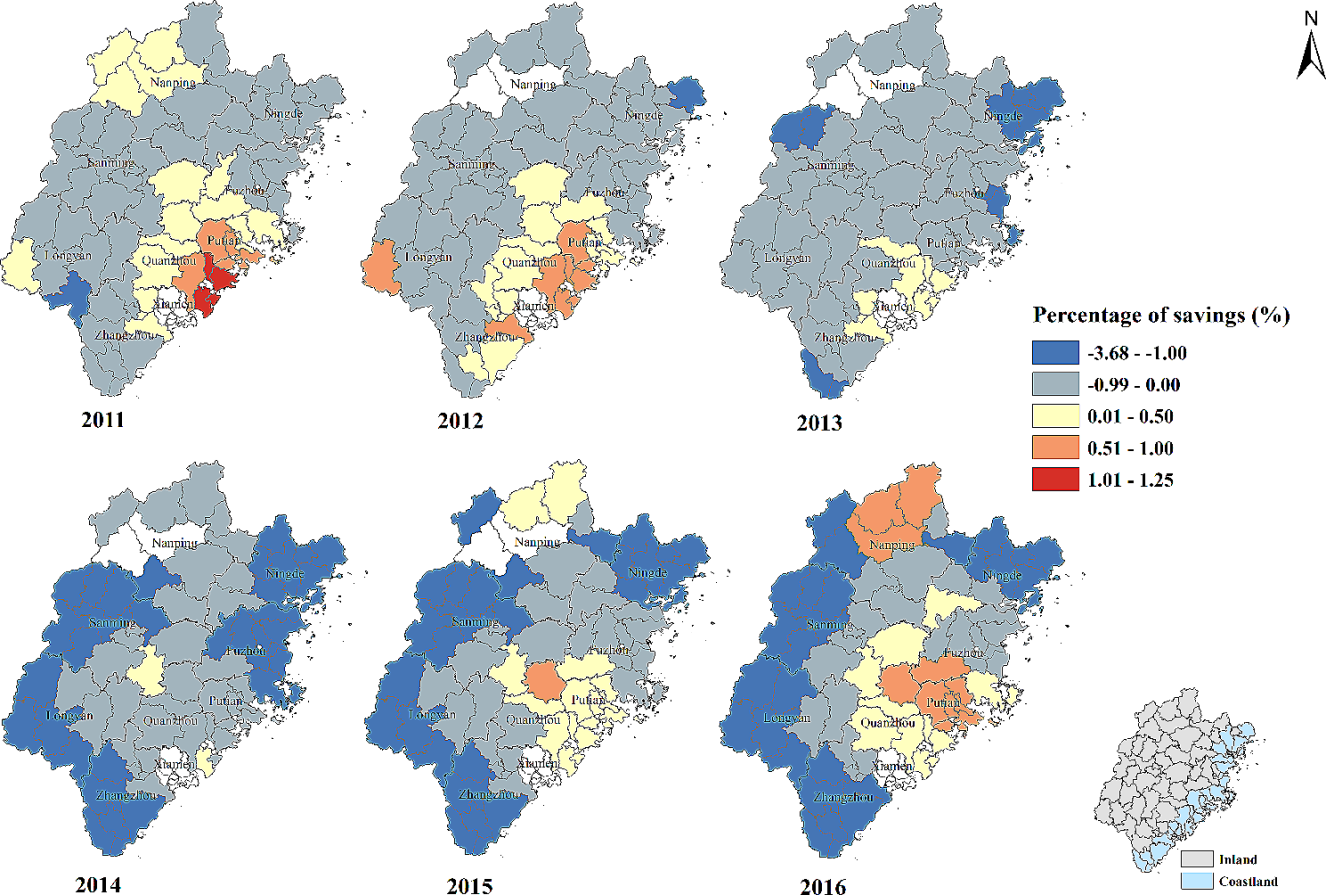
Temporal-spatial distribution of regression coefficients for “Percentage of savings (%)” in geographically and temporally weighted regression model. White color: no data

**Fig. 9:**
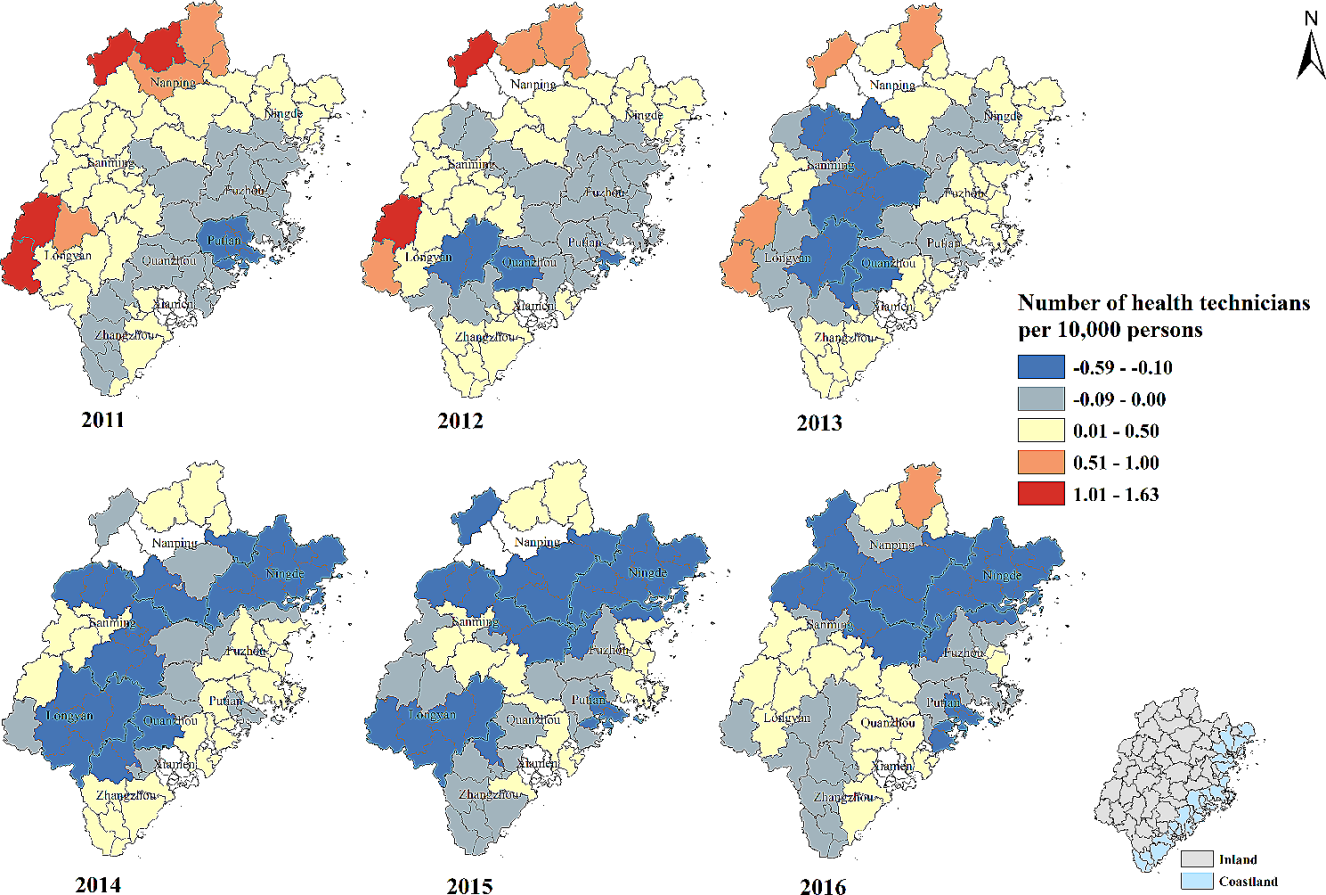
Temporal-spatial distribution of regression coefficients for “Number of health technicians per 10,000 persons” in geographically and temporally weighted regression model. White color: no data

The VIFs of seven explanatory variables were all less than 2, indicating that there was no multicollinearity among socioeconomic factors (Table 2). The GTWRM calculated regression coefficients of each explanatory variable in 77 counties from 2011 to 2016. The adjusted R square of GTWRM was higher than that of GLM (0.898 vs. 0.324). The statistics and temporal-spatial distribution characteristics of regression coefficients were displayed in Table 2; Figs. 3, 4, 5, 6, 7, 8 and 9.

**Table 2:** Statistics of regression coefficient in general linear model and geographically and temporally weighted regression model

Population	Variable	GLM				GTWRM						
		β (95%CI)	P	VIF		Mean	SD	Min	P25	Median	P75	Max
All	Percentage of female patients (%)	-1.02 (-1.5~-0.54)	< 0.001	1.04		-0.36	1.70	-4.54	-1.38	-0.32	0.53	8.97
	Percentage of patients who aged ≥ 60 years (%)	-0.75 (-1.11~-0.4)	< 0.001	1.25		-0.38	0.89	-3.51	-0.85	-0.45	0.04	2.95
	Percentage of low-income patients (%)	-2.86 (-4.04~-1.68)	< 0.001	1.11		-1.14	2.50	-6.50	-2.71	-1.51	-0.16	7.98
	GDP per capita (10,000 yuan per capita)	2.63 (1.48 ~ 3.77)	< 0.001	1.64		1.22	2.78	-17.92	0.05	1.17	3.01	7.26
	Carbon emission intensity (ton per 10,000 yuan)	-21.96 (-27.59~-16.34)	< 0.001	1.35		-12.07	14.29	-64.46	-19.00	-8.97	-1.60	16.79
	Percentage of savings (%)	-0.77 (-1.04~-0.5)	< 0.001	1.13		-0.52	0.80	-3.68	-0.96	-0.38	-0.03	1.25
	Number of HT per 10,000 persons	-0.12 (-0.23~-0.01)	0.031	1.32		0.01	0.26	-0.59	-0.11	-0.01	0.07	1.63
Outpatients	Percentage of female patients (%)	-0.07 (-0.54 ~ 0.41)	0.781	1.12		-0.13	1.19	-4.06	-0.81	-0.02	0.60	3.15
	Percentage of patients who aged ≥ 60 years (%)	0.34 (-0.02 ~ 0.70)	0.064	1.43		0.34	0.98	-3.04	-0.12	0.25	0.86	3.24
	Percentage of low-income patients (%)	-1.47 (-2.89~-0.06)	0.042	1.14		-0.67	3.03	-10.58	-2.55	-0.81	0.68	9.83
	GDP per capita (10,000 yuan per capita)	2.35 (0.99 ~ 3.72)	0.001	1.65		0.61	3.07	-16.76	-0.96	0.65	2.75	8.28
	Carbon emission intensity (ton per 10,000 yuan)	-19.73 (-26.58~-12.87)	< 0.001	1.42		-12.59	19.57	-101.12	-23.40	-8.70	-0.19	26.09
	Percentage of savings (%)	-0.66 (-0.98~-0.34)	< 0.001	1.14		-0.39	0.98	-4.37	-1.00	-0.28	0.27	1.93
	Number of HT per 10,000 persons	-0.10 (-0.23 ~ 0.03)	0.137	1.33		0.06	0.33	− 0.56	-0.12	-0.01	0.12	2.21

CI: confidence interval; GDP: Gross Domestic Product; GLM: general linear model; GTWRM: Geographically and temporally weighted regression model; HT: health technicians; Max: Maximum; Min: Minimum; SD: standard deviation; P25: Percentile 25; P75: Percentile 75; VIF: variance inflation factor

#### Percentage of female patients (%)

In general, the percentage of female patients and the percentage of patients whose visiting frequency ≥ 4 times were negatively correlated. The strength of negative correlation increased in Quanzhou, Longyan and Sanming city from 2011 to 2013. The strength of negative correlation gradually decreased and the number of counties with positive association was increasing from 2014 to 2016. The strong positive effect was found in some counties of Nanping and Zhangzhou city (Table 2; Fig. 3).

#### Percentage of patients who aged ≥ 60 years (%)

On the whole, the association between percentage of patients who aged ≥ 60 years and the percentage of patients whose visiting frequency ≥ 4 times showed negative correlation. The strength of negative correlation increased in some counties of Sanming, Nanping and Ningde city over time. In contrast, the patients who aged ≥ 60 years in Longyan city tended to see a doctor at least 4 times within a year and the positive effect was also gradually found in Quanzhou and Zhangzhou city from 2011 to 2016 (Table 2; Fig. 4).

#### Percentage of low-income patients (%)

Over all, the percentage of low-income patients and the percentage of patients whose visiting frequency ≥ 4 times were negatively correlated. The strength of negative correlation increased in Nanping, Ningde, Fuzhou, Putian and Quanzhou city from 2011 to 2016. Conversely, the positive association between percentage of low-income patients and the percentage of patients whose visiting frequency ≥ 4 times was found in Zhangzhou, Longyan and Sanming city over the six-year study period (Table 2; Fig. 5).

#### GDP per capita (10,000 yuan per capita)

The mean and median of regression coefficient were both greater than zero, implying that overall with the increase of GDP per capita, the percentage of patients whose visiting frequency ≥ 4 times increased. The strength of positive correlation increased in some counties of Putian, Quanzhou, Longyan, Sanming, Nanping and Ningde city from 2011 to 2016. On the other hand, there were negative association between GDP per capita and the percentage of patients whose visiting frequency ≥ 4 times in western, northern and eastern areas from 2011 to 2016. However, the number of counties with negative association was decreasing with time (Table 2; Fig. 6).

#### Carbon emission intensity (ton per 10,000 yuan)

There was positive association between carbon emission intensity and the percentage of patients whose visiting frequency ≥ 4 times in most of counties in 2011. The number of counties with positive association was decreasing from 2011 to 2015 and the strong negative effect was found in Zhangzhou, Sanming, Nanping and Ningde city in 2016 (Table 2; Fig. 7).

#### Percentage of savings (%)

In general, the percentage of savings and the percentage of patients whose visiting frequency ≥ 4 times were negatively correlated. The strength of negative correlation increased in some counties of Ningde, Zhangzhou, Longyan and Sanming city from 2011 to 2016. Nevertheless, the positive association was found in some counties of Putian, Quanzhou and Nanping city in the study period (Table 2; Fig. 8).

#### Number of HT per 10,000 persons

The regression coefficients of number of HT per 10,000 persons varied greatly in the same county at different time. The positive association between number of HT per 10,000 persons and the percentage of patients whose visiting frequency ≥ 4 times was found in most counties in 2011. The number of counties with negative association was increasing from 2011 to 2016 and such counties were spread throughout the cities of Fujian Province (Table 2; Fig. 9).

### Sensitivity analysis

Overall, the percentage of outpatients whose visiting frequency ≥ 4 times within a year was higher than that of all patients (Supplementary Table 2). However, the temporal-spatial distribution characteristics of the percentage were similar with that of all patients (Supplementary Fig. 3). There was spatial autocorrelation for the percentage between 2012 and 2016 (Supplementary Table 3). The percentage was presented low-low cluster in Ningde city and high-high cluster in Quanzhou city during the study period. While the local spatial autocorrelation changes from low-low cluster to high-high cluster in Sanming city (Supplementary Fig. 4). The adjusted R square of GTWRM for the associations between seven socioeconomic factors and percentage of outpatients whose visiting frequency ≥ 4 times within a year was 0.873. Except for the percentage of patients who aged ≥ 60 years, the statistics and temporal-spatial distribution characteristics of regression coefficients of socioeconomic factors were also similar with that of all patients (Table 2 and Supplementary Fig. 5 ─ 11). As a whole, there was positive association between percentage of outpatients who aged ≥ 60 years and the percentage of outpatients whose visiting frequency ≥ 4 times within a year. The number of counties with positive association was increasing from 2011 to 2016. The strong positive effect was found in Quanzhou, Longyan and Sanming city over time. (Table 2 and Supplementary Fig. 6).

## Discussion

Regular follow-up and medication can effectively reduce the risk of cardiovascular diseases, cerebrovascular diseases and kidney diseases for patients with hypertension [22]. However, the individual-level visiting data of rural patients with hypertension in Fujian province from 2011 to 2016 showed that the visiting rate was low. Among the patients who saw a doctor, the percentage of patients whose visiting frequency ≥ 4 times within a year gradually increased with time, but the percentage was still less than 80% by 2016. The associations between percentage of patients whose visiting frequency ≥ 4 times within a year and socioeconomic factors had temporal-spatial heterogeneity.

The visiting rate of rural patients with hypertension of all ages gradually increased from 2011 to 2016, but it was still far less than the prevalence rate of hypertension for Chinese rural adult residents (3.43% VS 23.2%) [4]. The reasons for the large gap may be as follows: First, the rural residents with NRCMS under 18 years old were included in this study whose prevalence rate of hypertension was relatively low; Second, the diagnosis of hypertension in this study was more rigorous, which was defined as an average systolic BP ≥ 140 mmHg and/or diastolic BP ≥ 90 mmHg measured three times on different days. In the random sampling screening study, BP was the average of three measurements with 30 s between each measurement. Third, “hypertension” was used as the only searching term. The patients who were only given a diagnosis of the complication rather than hypertension were not included in this study. Fourth, a significant proportion of rural residents were unaware of having high BP, or some rural residents were aware of having high BP but did not seek medical treatment to control BP.

This study found that the proportion of outpatients increased while the proportion of inpatients decreased and the outpatients shown higher percentage of visiting frequency ≥ 4 times within a year than inpatients. The per capita total medical expenditures decreased over time. It implied that increasing outpatient utilization might reduce the risk of hospitalization and disease burden, and further reflected the importance of health management. Therefore, effective measures should be taken to improve the health service utilization, especially the outpatient service utilization for rural patients with hypertension. Furthermore, out-of-pocket medical expenditures had significantly reduced from 2011 to 2016, but the top 10% of patients with the highest medical expenditures spent 45% of the total cost of all patients. It indicated that the distribution of medical expenditures was very uneven. Effective management mode should be developed to reduce unnecessary and costly treatment and improve the equity of health service utilization. The patients with high medical expenditures who were mainly inpatients and elderly should be paid more attention to.

Follow-up helps to timely detect the changes of patients’ condition and know whether the patient obeys the doctor’s advice, thereby providing targeted health guidance and intervention. The visiting frequency is an important indicator to assess the development and utilization of follow-up work and affects the effect of follow-up and patients’ satisfaction. Peng et al. found that there were positive effects of service content, frequency, mode, and institutions of follow-up management on the health outcomes for patients with hypertension [23]. It was suggested that the patients who achieved targeted BP level should see a doctor at least 4 times and the patients who did not achieve should be followed up 12 or more times within a year [1, 18]. However, the visiting frequency of the patients with hypertension who saw a doctor was suboptimal. Although the visiting frequency gradually increased over time during the six-year study period, still less than 80% of patients were followed up at least four visits within a year.

Geographically, the likelihood of visiting frequency ≥ 4 times within a year for the patients lived in northern and eastern areas of Fujian province was even lower. This study found that the percentage of female patients, percentage of patients who aged ≥ 60 years, percentage of low-income patients, GDP per capita were negatively related to the percentage of patients whose visiting frequency ≥ 4 times within a year in the above areas. It implied that female, ageing and low-income limited the regular follow-up of patients with hypertension, even if economic development could not improve this situation. Inversely, the percentage of female patients, percentage of patients who aged ≥ 60 years and percentage of low-income patients were positively correlated with the percentage of patients whose visiting frequency ≥ 4 times within a year in Sanming, Longyan, Zhangzhou and/or Quanzhou city. “Sanming model” was put forward to systematically reform in the governance structure, payment system and physician compensation methods in 2013 and achieved some results [24–26]. The medical insurance fund had a balance of about 100 million yuan by 2015. It promoted Sanming government to provide 10 kinds of free drugs for outpatients with hypertension in medical institutions below the county level. Thus, the percentage of patients whose visiting frequency ≥ 4 times within a year was markedly increased since 2015. The female, elderly and low-income patients were stimulated to follow up to a large extent in Sanming city. Although Longyan, Zhangzhou and Quanzhou government did not take additional steps to strengthen the health management of hypertension, the female, elderly and low-income patients in these areas were also more likely to see a doctor at least 4 times within a year. National Healthcare Security Administration and Health Commission put forward special action plans to ensure outpatient medication and health management for hypertension and diabetes in 2020 [27]. Longyan and Quanzhou city were chosen as exemplary cities to explore innovative models and typical experiences which would be popularized and replicated to other cities.

Carbon emission intensity is an environmental evaluation index which measures the relationship between carbon dioxide emissions and economic development of a country or region [28]. The smaller the carbon emission intensity, the higher the energy utilization efficiency of the region. Excessive carbon emissions lead to the destruction of the ozone layer, which causes global warming and extreme weather. Fujian province located in the southeast of China of which the average annual temperature was around 20 ℃ and the daily maximum temperature exceeded 40 ℃. The temperature had been increasing over time. Our previous study found that high carbon emission intensity increased hospital admissions of cardiovascular diseases for rural residents [29]. It was attributed to the high temperature which induced serious cardiovascular disease and increased the mortality [30]. Conversely, this study shown that there was negative association between carbon emission intensity and the percentage of patients whose visiting frequency ≥ 4 times within a year in most of counties. Unlike hospitalization, the follow-up intervals were relatively flexible in the absence of life-threatening conditions. Patients with hypertension were reluctant to go out in extreme weather which may be one of important reasons for limiting the regular follow-up. Therefore, it was necessary to balance the relationship between economic development and the environment. Reducing carbon emissions without damaging economic growth would both be beneficial for reducing hospitalization and increasing follow-up.

This study also found negative relationship between the percentage of patients whose visiting frequency ≥ 4 times within a year and percentage of savings in most of counties. It could be explained that the less the visiting frequency, the lower the economic burden caused by seeking medical treatments, the higher the savings. The consumption habits of Chinese residents had a certain lag. They were used to saving before consumption. Although medical insurance system reduced the out-of-pocket expenses to a great extent, the transportation expenses, lost income and time cost generated by seeking medical treatments could not be avoided. Thus, patients tended to save rather than consume in the absence of life-threatening conditions. Besides, the increasing number of HT per 10,000 persons did not increase the likelihood of visiting frequency ≥ 4 times within a year for patients with hypertension. Instead, the negative association between number of HT per 10,000 persons and the percentage of patients whose visiting frequency ≥ 4 times within a year was found from 2014 to 2016, especially in northern and eastern areas of Fujian province. It implied that existing health resources were not reasonably allocated to play positive role in increasing the health service utilization.

To sum up, multiple socioeconomic factors affected the visiting frequency for rural patients with hypertension. The relevant prevention and control measures need to start from multiple aspects to improve the follow-up compliance of patients. For the whole province, the government is suggested to regularly organize free clinic to improve the health awareness of residents and energetically promote the importance of health management for chronic diseases. Female and elderly residents should be the focus groups for publicity and education. Different modes of providing health management services should be developed and the family doctor services should be effectively put into effect. For Nanping, Ningde, Fuzhou, Putian and Quanzhou city, providing several kinds of free drugs for low-income patients may promote their follow-up compliance to a large extent. For Nanping, Ningde, Zhangzhou and Sanming city, improving energy utilization efficiency can be considered to reduce carbon emissions, so as to improve the follow-up compliance of patients. For Nanping and Ningde city, health resources should be reasonably allocated to improve the efficiency and quality of health service.

Some limitations of our study must be acknowledged. First, the database of NRCMS only contained the medical information of outpatients and inpatients. Follow-up records by home visit and telephone were not included in this study limited by data source. Second, the complications of hypertension were complex and the search terms were difficult to be comprehensive. “Hypertension” as the only searching term may result in underestimating the visiting frequency. Third, the visiting frequency was counted by natural year and some patients were diagnosed as hypertension at the end of the year which may underestimate their visiting frequency within a year. Fourth, the results only reflected the situations of study period (2011–2016) which was limited by the availability of latest data. We are actively applying for access to the latest data and will update the perceptions in the future. Last, there may be the risk of ecological fallacies as this study was conducted at the county level. Individual-level data was needed to validate the findings.

## Conclusions

The visiting rate and the visiting frequency within a year for rural patients with hypertension in Fujian province was suboptimal. There was temporal-spatial heterogeneity in the associations between percentage of patients whose visiting frequency ≥ 4 times within a year and socioeconomic factors. Female and elderly patients should be the focus of health management. Effectively implementing the family doctor services, providing several kinds of free antihypertensive drugs, improving energy utilization efficiency and reasonably allocating the health resources may be the effective strategies to improve the follow-up compliance of patients.

### Electronic supplementary material

Below is the link to the electronic supplementary material.

Supplementary Material 1

## Data Availability

The datasets used in this study are available from the corresponding author on reasonable request.
