# Supplementary material for: Temporal-spatial distribution characteristics and associated socioeconomic factors of visiting frequency for rural patients with hypertension in Fujian Province, Southeast China
Source: BMC Public Health. 2024 Mar 1;24:656. doi: 10.1186/s12889-024-18113-9 (PMC10905804; doi:10.1186/s12889-024-18113-9)

Supplementary Table 1 Visiting rate of rural patients with hypertension in Fujian province from 2011 to 2016

| Year | Number of patients | Rural resident population (10 thousand) ^a^ | Participation rate of NRCMS (%) ^a^ | Number of participants in NRCMS (10 thousand) ^b^ | Visiting rate ^c^ (%) |
| --- | --- | --- | --- | --- | --- |
| 2011 | 113,192 | 1,559 | 92.11 | 1,436 | 0.79 |
| 2012 | 182,541 | 1,514 | 94.74 | 1,434 | 1.27 |
| 2013 | 257,578 | 1,481 | 93.15 | 1,380 | 1.87 |
| 2014 | 321,892 | 1,454 | 96.80 | 1,407 | 2.29 |
| 2015 | 388,889 | 1,436 | 97.31 | 1,397 | 2.78 |
| 2016 | 473,341 | 1,410 | 97.94 | 1,381 | 3.43 |

^a^: Extracted from Fujian Provincial Bureau of Statistics (Accessible at https://tjj.fujian.gov.cn/xxgk/ndsj/); ^b^: “Number of participants in NRCMS” was equal to “rural resident population” times “participation rate of NRCMS”; ^c^: “Visiting rate” was equal to “number of patients” divided by “number of participants in NRCMS”.

Supplementary Table 2 Statistics of dependent and explanatory variables in geographically and temporally weighted regression model

| Population | Variable |  | Mean | SD | Minimum | Percentile 25 | Median | Percentile 75 | Maximum |
| --- | --- | --- | --- | --- | --- | --- | --- | --- | --- |
| All | Dependent variable | Percentage of visiting frequency ≥4 times (%) | 32.42 | 25.33 | 0.00 | 4.51 | 34.31 | 55.43 | 81.12 |
|  | Explanatory variable | Percentage of female patients (%) | 59.76 | 4.15 | 48.15 | 56.90 | 60.27 | 62.76 | 75.82 |
|  |  | Percentage of patients who aged ≥60 years (%) | 65.77 | 6.20 | 51.02 | 61.91 | 65.02 | 69.10 | 91.56 |
|  |  | Percentage of low-income patients (%) | 2.16 | 1.76 | 0.00 | 0.63 | 1.94 | 3.32 | 11.43 |
|  |  | GDP per capita (10,000 yuan per capita) | 5.55 | 2.20 | 1.68 | 4.02 | 5.21 | 6.53 | 16.84 |
|  |  | Carbon emission intensity (ton per 10,000 yuan) | 1.15 | 0.41 | 0.35 | 0.86 | 1.11 | 1.40 | 2.66 |
|  |  | Percentage of savings (%) | 24.61 | 7.70 | 5.73 | 19.44 | 24.73 | 30.11 | 43.34 |
|  |  | Number of health technicians per 10,000 persons | 46.50 | 20.34 | 14.40 | 32.93 | 43.21 | 53.68 | 140.57 |
| Outpatients | Dependent variable | Percentage of visiting frequency ≥4 times (%) | 39.84 | 28.20 | 0.00 | 7.95 | 44.80 | 63.83 | 86.09 |
|  | Explanatory variable | Percentage of female patients (%) | 58.30 | 5.22 | 33.33 | 54.66 | 58.63 | 62.09 | 72.58 |
|  |  | Percentage of patients who aged ≥60 years (%) | 63.20 | 7.78 | 38.14 | 58.68 | 63.07 | 67.68 | 100.00 |
|  |  | Percentage of low-income patients (%) | 2.02 | 1.76 | 0.00 | .56 | 1.88 | 3.07 | 13.64 |
|  |  | GDP per capita (10,000 yuan per capita) | 5.55 | 2.20 | 1.68 | 4.02 | 5.21 | 6.53 | 16.84 |
|  |  | Carbon emission intensity (ton per 10,000 yuan) | 1.15 | 0.41 | 0.35 | 0.86 | 1.11 | 1.40 | 2.66 |
|  |  | Percentage of savings (%) | 24.61 | 7.70 | 5.73 | 19.44 | 24.73 | 30.11 | 43.34 |
|  |  | Number of health technicians per 10,000 persons | 46.50 | 20.34 | 14.40 | 32.93 | 43.21 | 53.68 | 140.57 |

GDP: Gross Domestic Product; SD: standard deviation.

Supplementary Table 3 Global spatial autocorrelation analysis of percentage of visiting frequency ≥4 times within a year for rural patients with hypertension in Fujian province from 2011 to 2016

| Population | Year | Moran's Index | Z | P |
| --- | --- | --- | --- | --- |
| All | 2011 | 0.0651 | 1.0698 | 0.2847 |
|  | 2012 | 0.0941 | 1.6561 | 0.0977 |
|  | 2013 | 0.3757 | 5.7827 | <.0001 |
|  | 2014 | 0.3637 | 5.6315 | <.0001 |
|  | 2015 | 0.3697 | 5.7616 | <.0001 |
|  | 2016 | 0.4196 | 5.2968 | <.0001 |
| Outpatients | 2011 | 0.0496 | 1.0024 | 0.3161 |
|  | 2012 | 0.1438 | 2.3655 | 0.0180 |
|  | 2013 | 0.3682 | 5.6714 | <.0001 |
|  | 2014 | 0.3482 | 5.4333 | <.0001 |
|  | 2015 | 0.3848 | 6.0382 | <.0001 |
|  | 2016 | 0.3404 | 4.3913 | <.0001 |

Supplementary Figure 1 Lorenz curve and Gini coefficient of total medical expenditures for hypertension from 2011 to 2016.


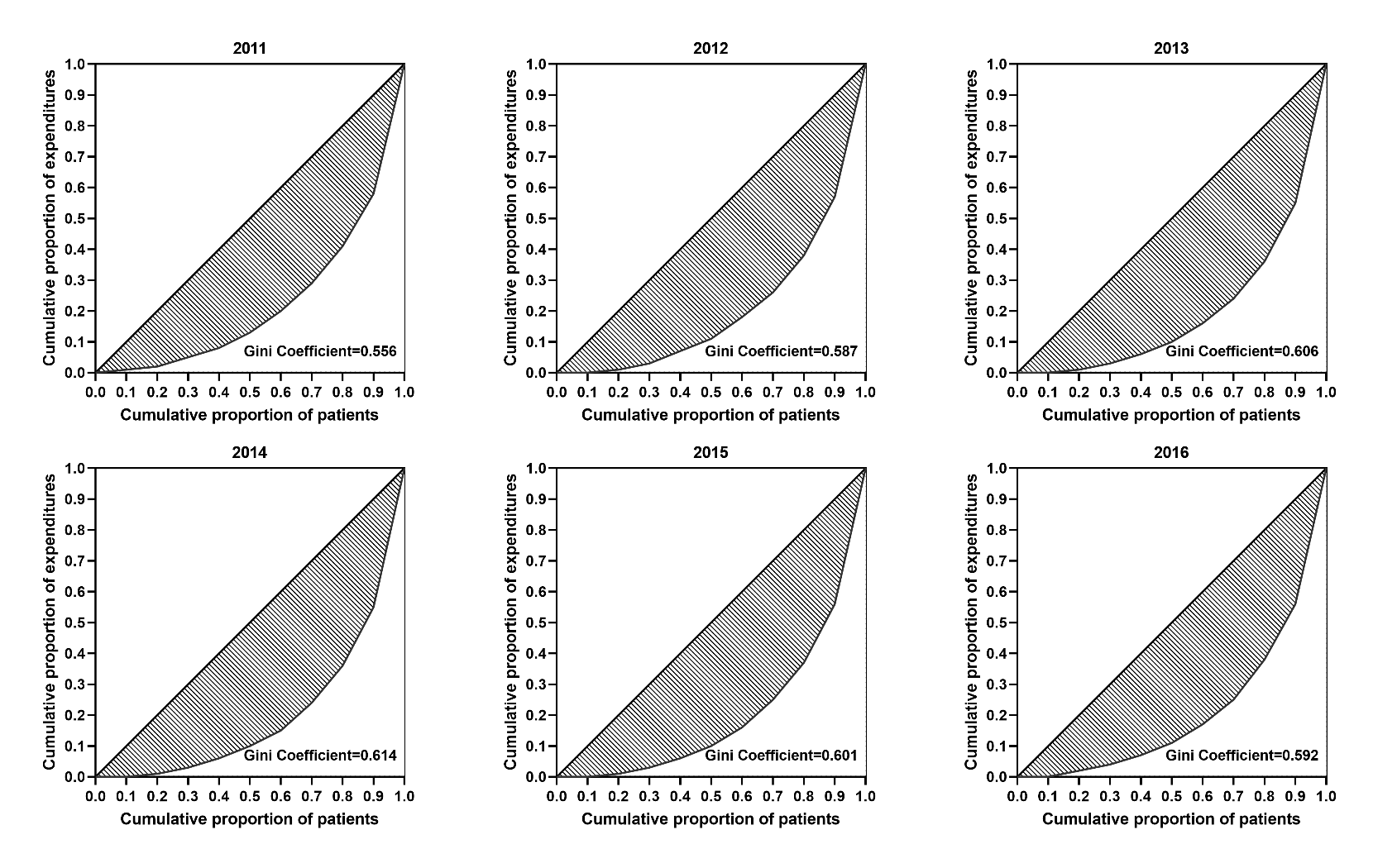


Supplementary Figure 2 Local spatial autocorrelation analysis of percentage of visiting frequency ≥4 times within a year for rural patients with hypertension in Fujian province from 2011 to 2016. White color: no data.


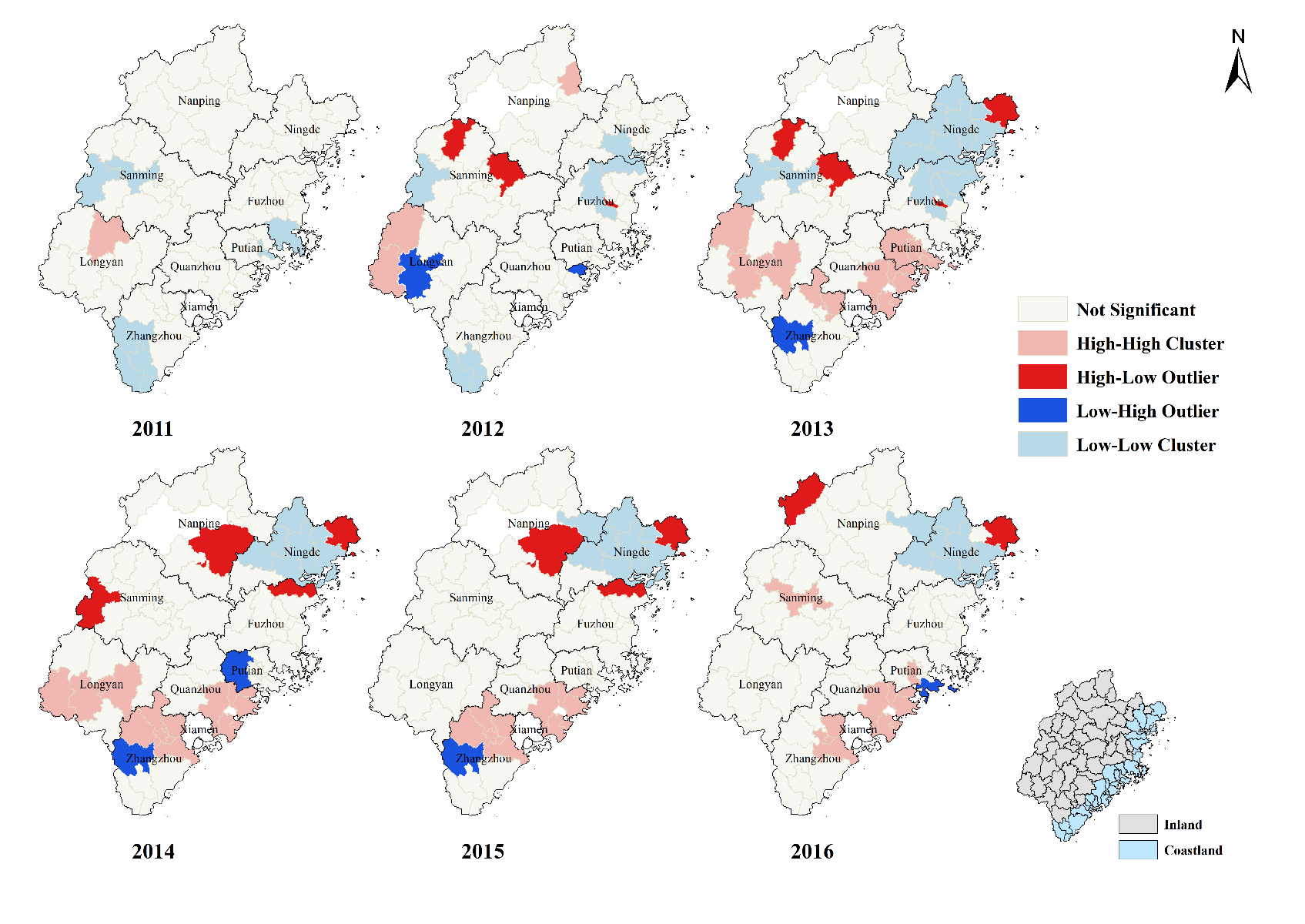


Supplementary Figure 3 Percentage of visiting frequency ≥4 times within a year for rural outpatients with hypertension in Fujian province from 2011 to 2016. The redder the color, the higher the percentage. White color: no data.


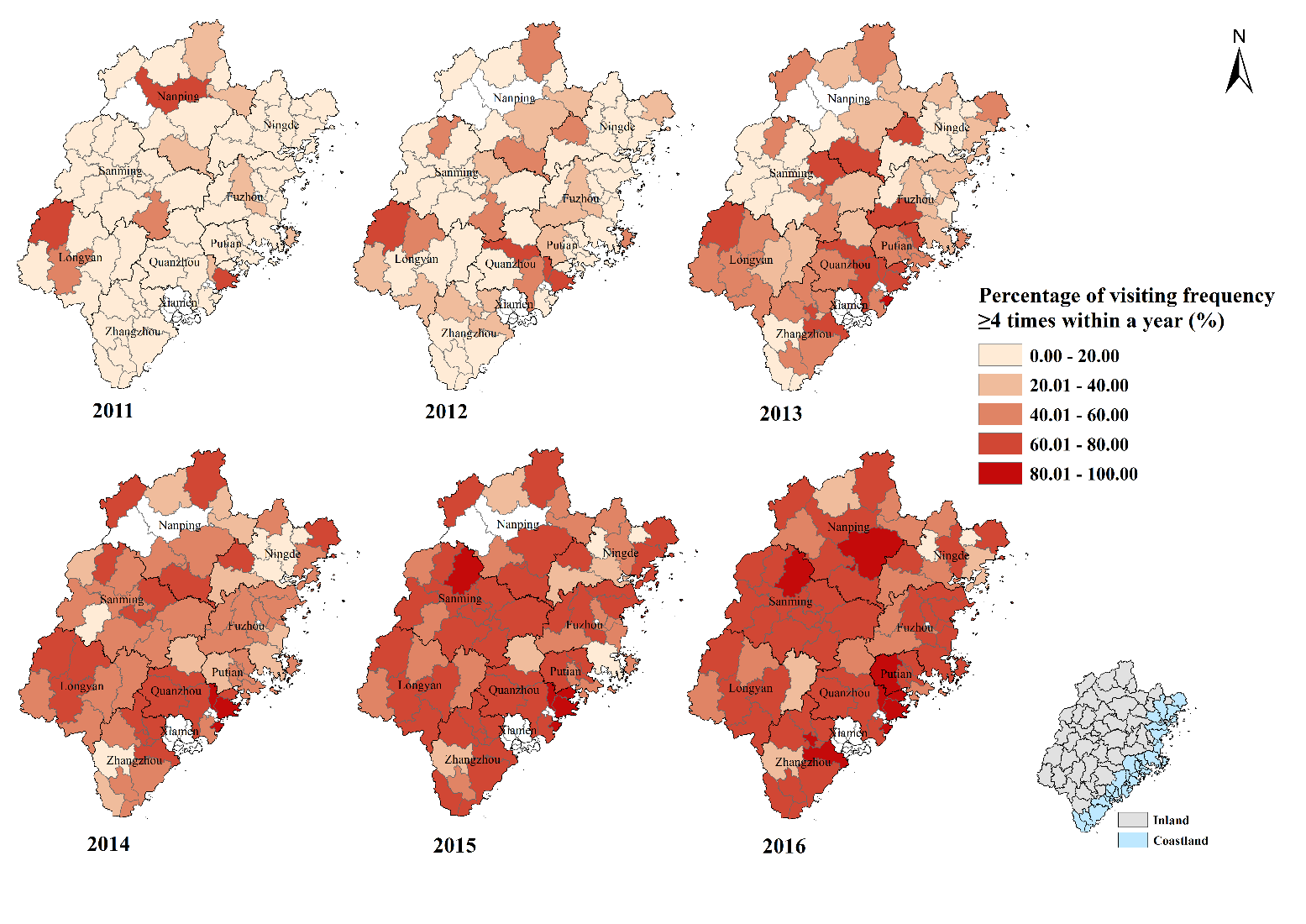


Supplementary Figure 4 Local spatial autocorrelation analysis of percentage of visiting frequency ≥4 times within a year for rural outpatients with hypertension in Fujian province from 2011 to 2016. White color: no data.


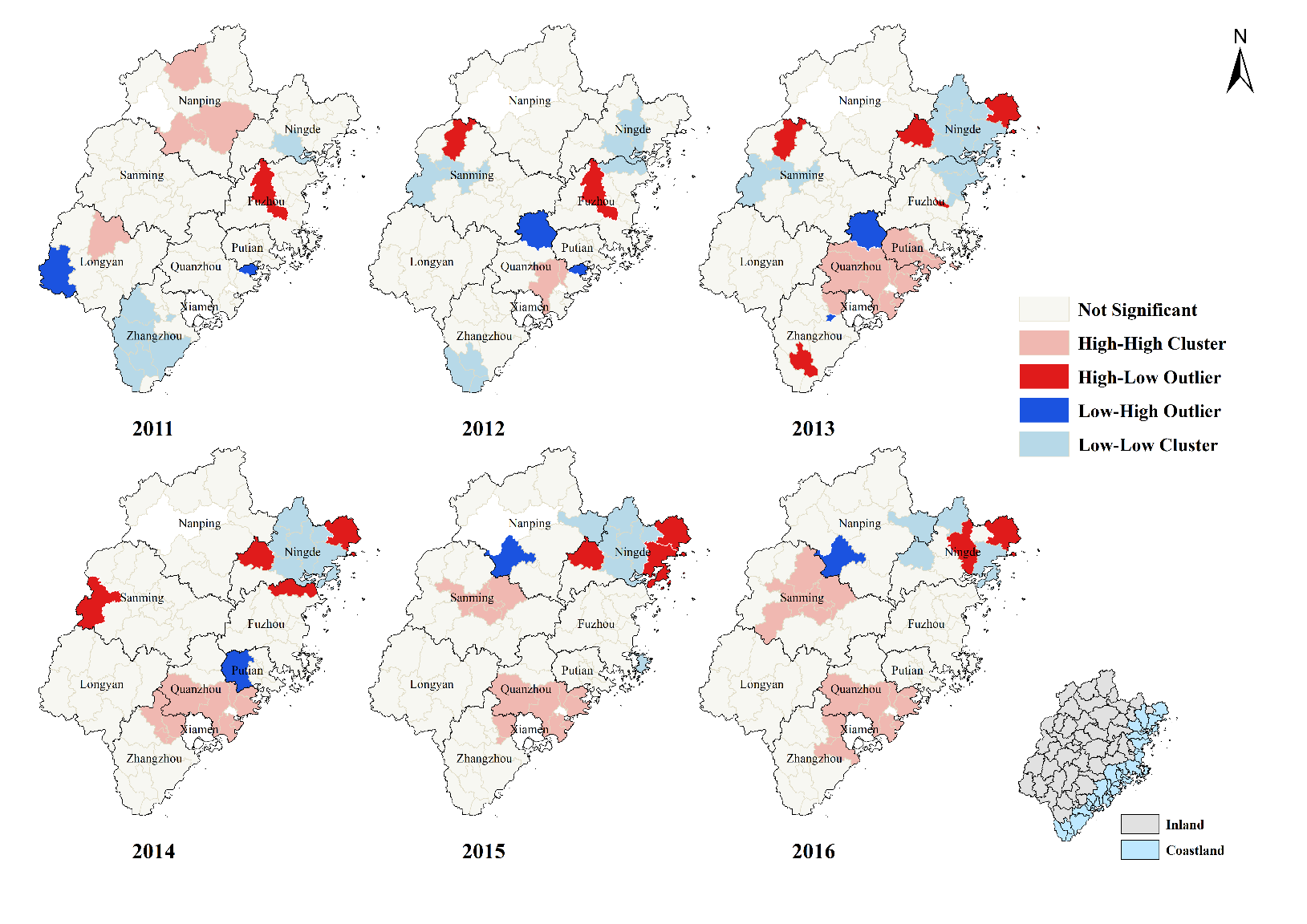


Supplementary Figure 5 Temporal-spatial distribution of regression coefficients for “Percentage of female patients (%)” in geographically and temporally weighted regression model among rural outpatients. White color: no data.


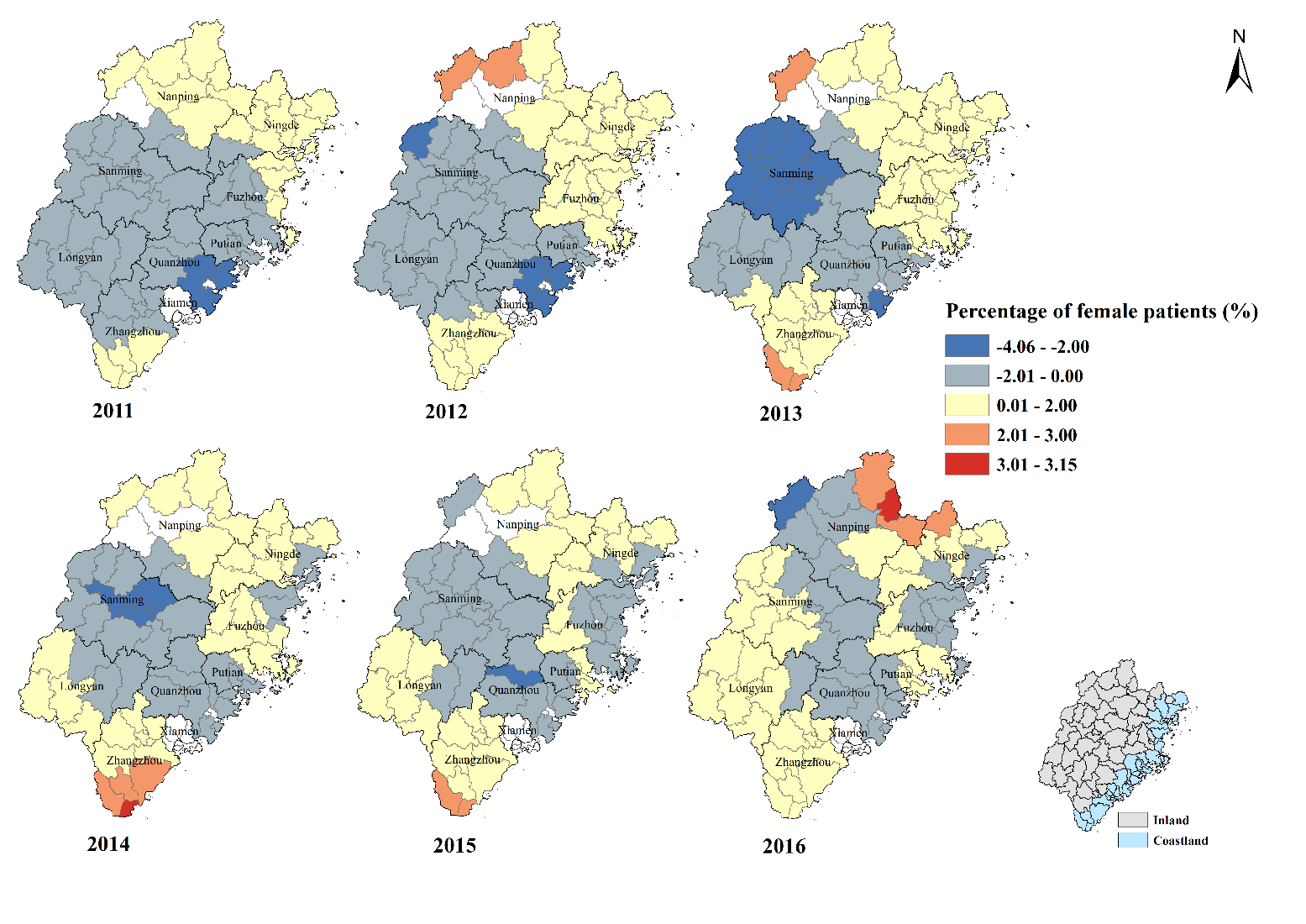


Supplementary Figure 6 Temporal-spatial distribution of regression coefficients for “Percentage of patients who aged ≥60 years (%)” in geographically and temporally weighted regression model among rural outpatients. White color: no data.


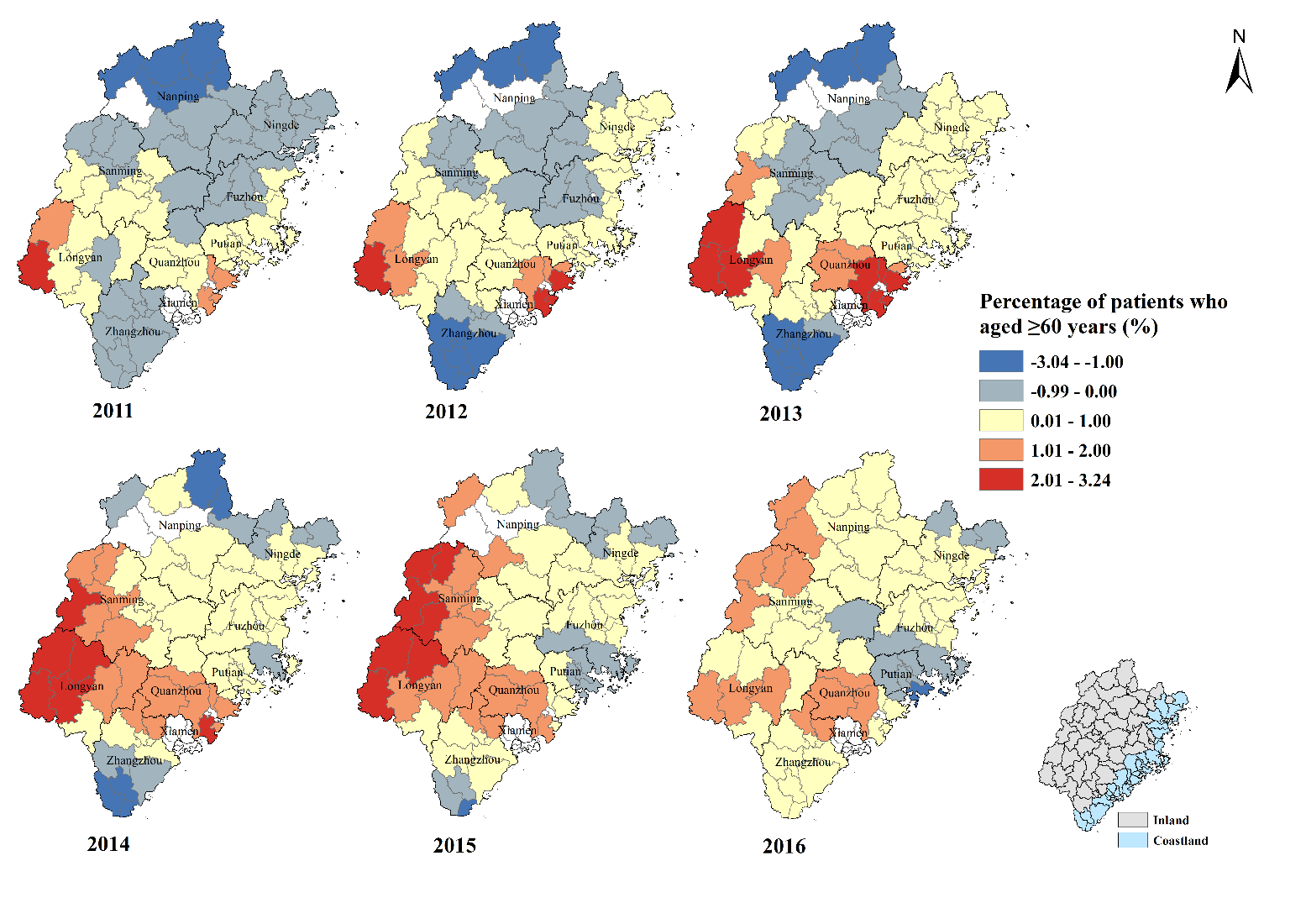


Supplementary Figure 7 Temporal-spatial distribution of regression coefficients for “Percentage of low-income patients (%)” in geographically and temporally weighted regression model among rural outpatients. White color: no data.


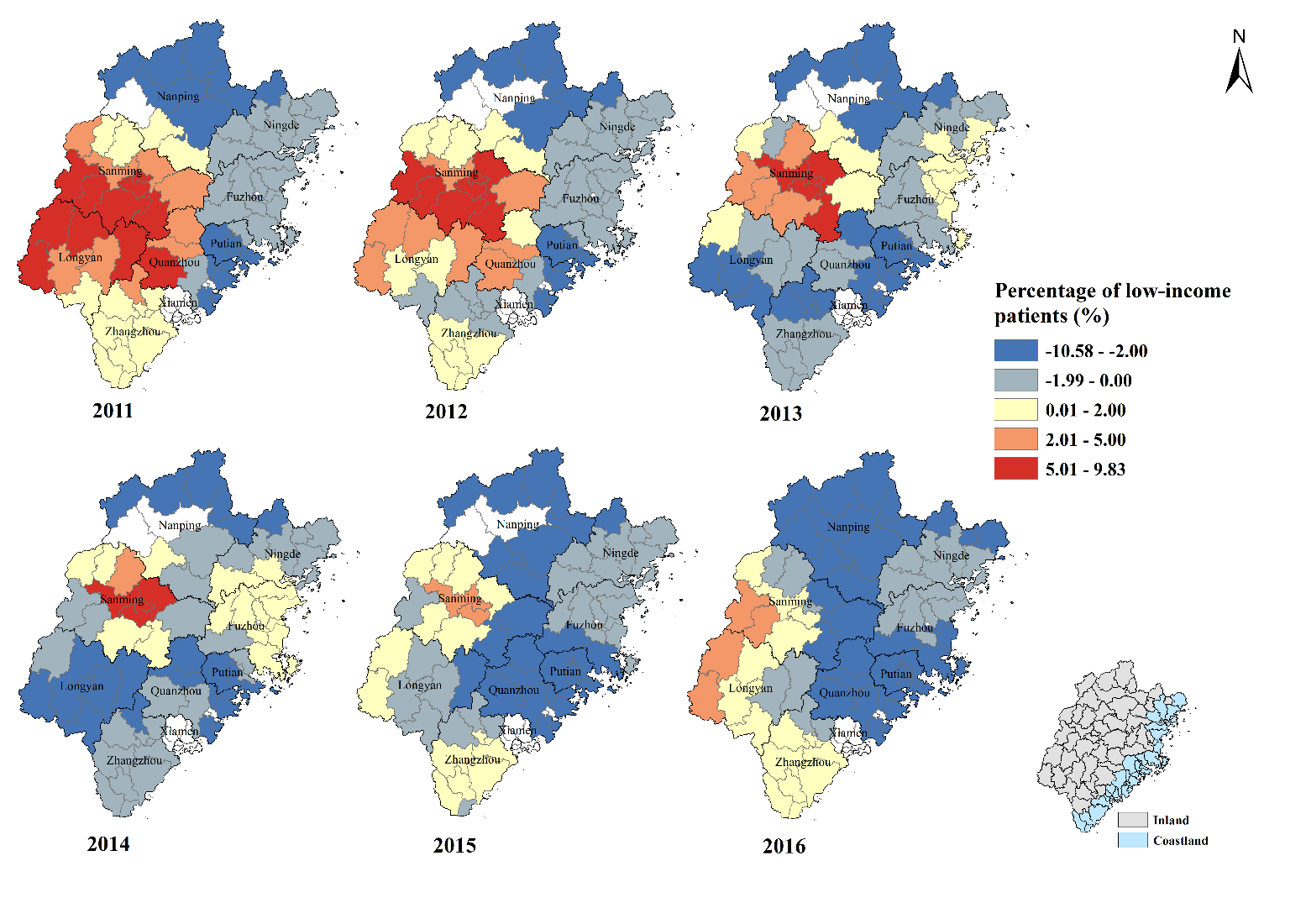


Supplementary Figure 8 Temporal-spatial distribution of regression coefficients for “GDP per capita (10,000 yuan per capita)” in geographically and temporally weighted regression model among rural outpatients. GDP: Gross Domestic Product. White color: no data.


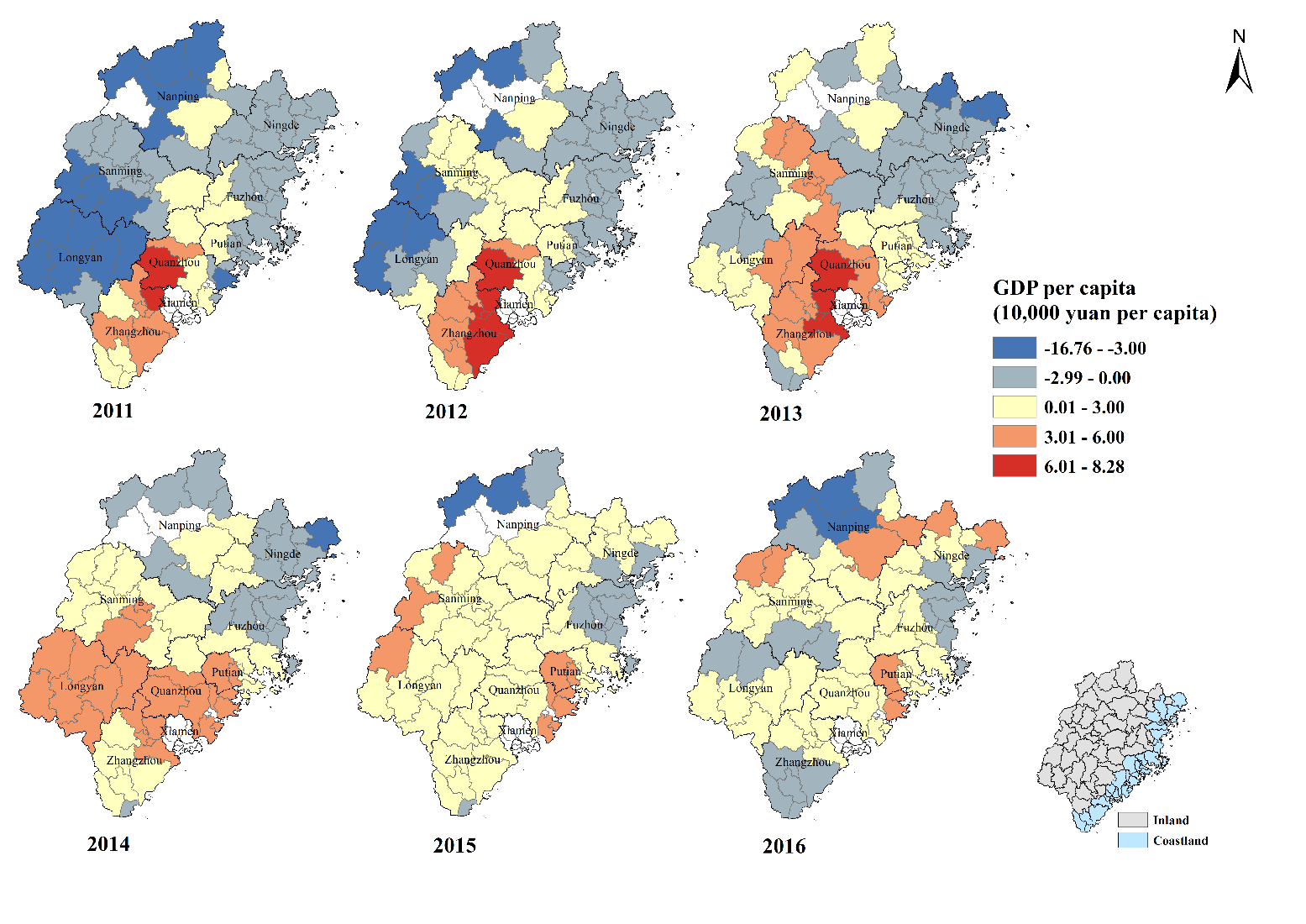


Supplementary Figure 9 Temporal-spatial distribution of regression coefficients for “Carbon emission intensity (ton per 10,000 yuan)” in geographically and temporally weighted regression model among rural outpatients. White color: no data.


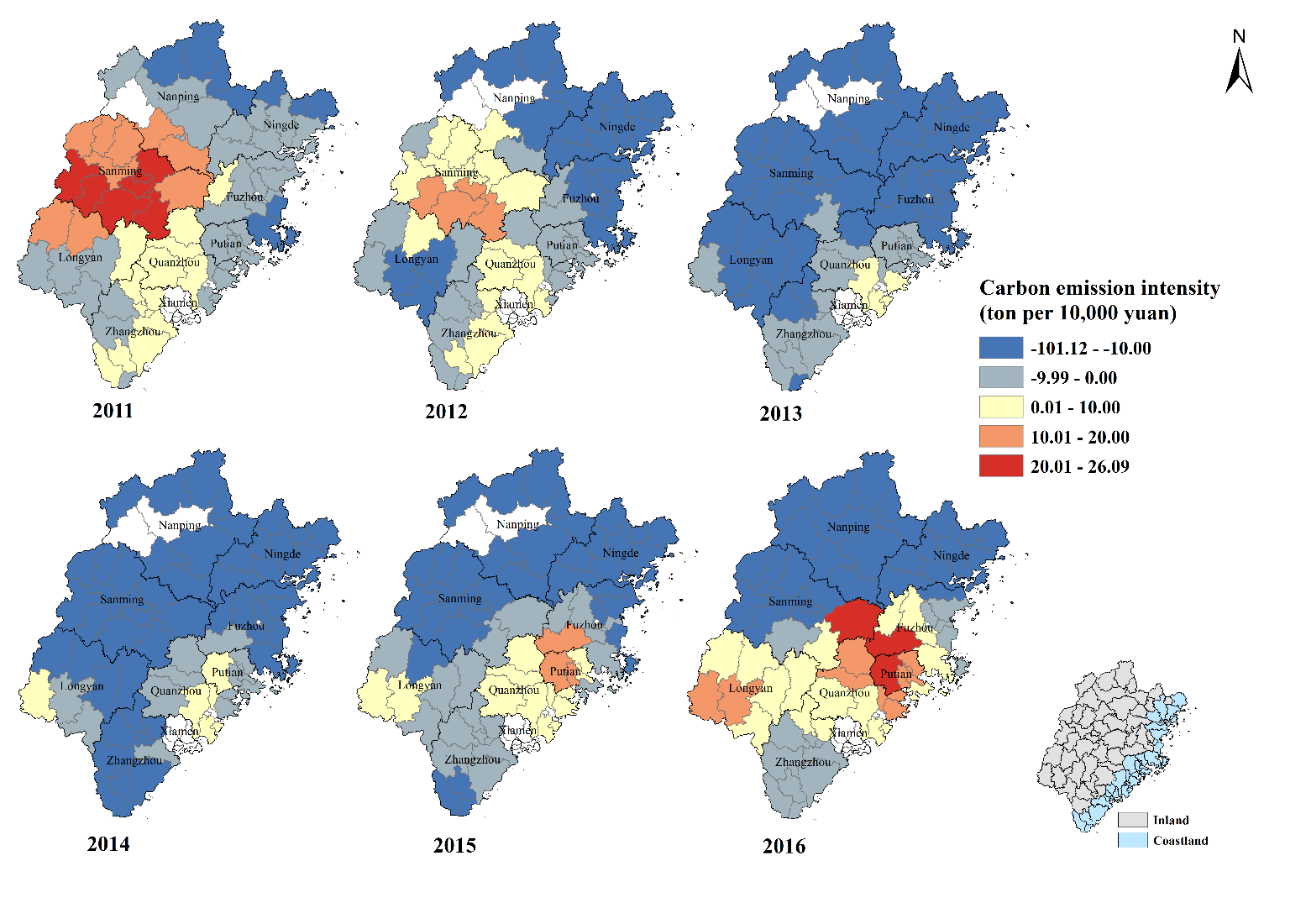


Supplementary Figure 10 Temporal-spatial distribution of regression coefficients for “Percentage of savings (%)” in geographically and temporally weighted regression model among rural outpatients. White color: no data.


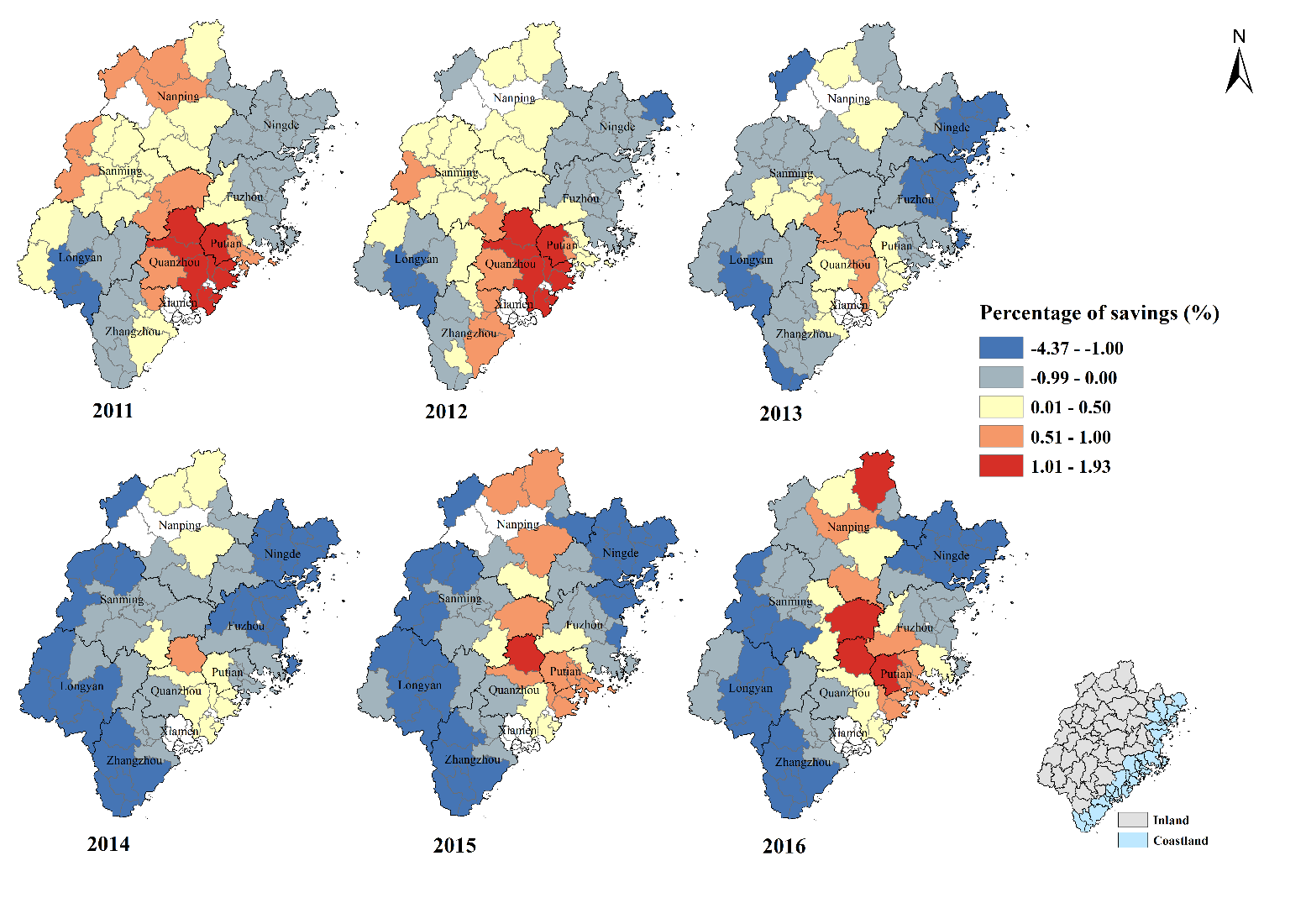


Supplementary Figure 11 Temporal-spatial distribution of regression coefficients for “Number of health technicians per 10,000 persons” in geographically and temporally weighted regression model among rural outpatients. White color: no data.


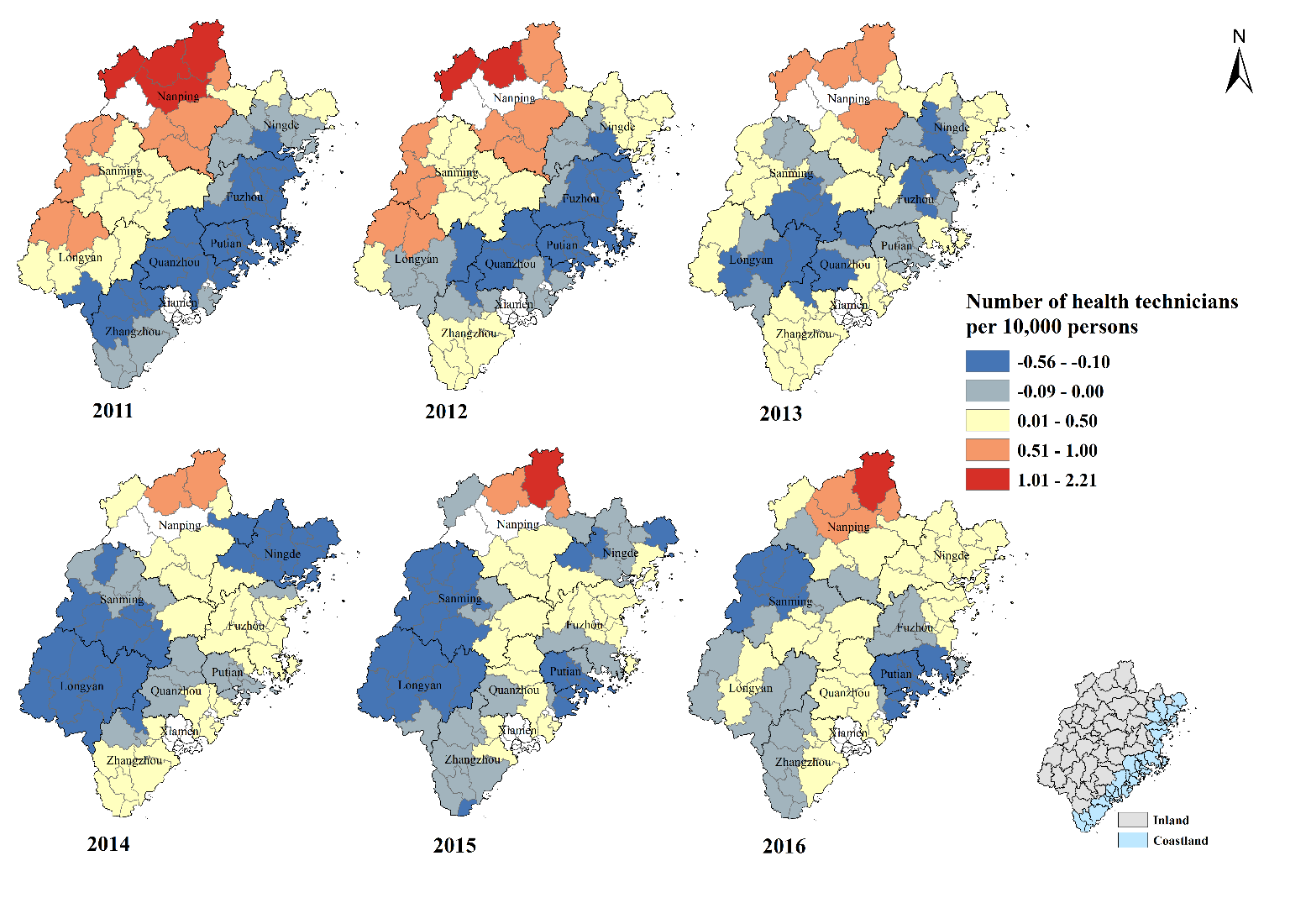

Supplement: Supplementary file 1 — Supplementary Material 1 [file 12889_2024_18113_MOESM1_ESM.docx]
